# Structural insights into the substrate recognition of serine palmitoyltransferase from *Sphingobacterium multivorum*

**DOI:** 10.1016/j.jbc.2023.104684

**Published:** 2023-04-07

**Authors:** Hiroko Ikushiro, Taiki Murakami, Aya Takahashi, Asuka Katayama, Taiki Sawai, Haruna Goto, Sajeer Koolath, Yuta Murai, Kenji Monde, Ikuko Miyahara, Nobuo Kamiya, Takato Yano

**Affiliations:** 1 Department of Biochemistry, Faculty of Medicine, Osaka Medical and Pharmaceutical University, Takatsuki, Osaka, Japan; 2 Department of Chemistry, Graduate School of Science, Osaka Metropolitan University, Osaka, Osaka, Japan; 3 Frontier Research Center for Advanced Material and Life Science, Faculty of Advanced Life Science, Hokkaido University, Sapporo, Hokkaido, Japan; 4 Research Center for Artificial Photosynthesis, Osaka Metropolitan University, Osaka, Osaka, Japan

**Keywords:** crystal structure, PLP-dependent enzyme, serine palmitoyltransferase, sphingolipid, X-ray crystallography

## Abstract

Serine palmitoyltransferase (SPT) is a key enzyme of sphingolipid biosynthesis, which catalyzes the pyridoxal-5′-phosphate–dependent decarboxylative condensation reaction of l-serine (l-Ser) and palmitoyl-CoA (PalCoA) to form 3-ketodihydrosphingosine called long chain base (LCB). SPT is also able to metabolize l-alanine (l-Ala) and glycine (Gly), albeit with much lower efficiency. Human SPT is a membrane-bound large protein complex containing SPTLC1/SPTLC2 heterodimer as the core subunits, and it is known that mutations of the SPTLC1/SPTLC2 genes increase the formation of deoxy-type of LCBs derived from l-Ala and Gly to cause some neurodegenerative diseases. In order to study the substrate recognition of SPT, we examined the reactivity of *Sphingobacterium multivorum* SPT on various amino acids in the presence of PalCoA. The *S. multivorum* SPT could convert not only l-Ala and Gly but also l-homoserine, in addition to l-Ser, into the corresponding LCBs. Furthermore, we obtained high-quality crystals of the ligand-free form and the binary complexes with a series of amino acids, including a nonproductive amino acid, l-threonine, and determined the structures at 1.40 to 1.55 Å resolutions. The *S. multivorum* SPT accommodated various amino acid substrates through subtle rearrangements of the active-site amino acid residues and water molecules. It was also suggested that non-active-site residues mutated in the human SPT genes might indirectly influence the substrate specificity by affecting the hydrogen-bonding networks involving the bound substrate, water molecules, and amino acid residues in the active site of this enzyme. Collectively, our results highlight SPT structural features affecting substrate specificity for this stage of sphingolipid biosynthesis.

Serine palmitoyltransferase (SPT) is a key enzyme of sphingolipid biosynthesis and catalyzes the pyridoxal-5′-phosphate (PLP)-dependent decarboxylative condensation reaction between l-serine (l-Ser) and palmitoyl-CoA (PalCoA) (1, 2) to form 3-ketodihydrosphingosine (KDS) (Fig. S1), alias long chain base (LCB), as a common precursor of all sphingolipids (1). Eukaryotic SPT functions as a membrane-bound large protein complex composed of SPTLC1/SPTLC2- or SPTLC1/SPTLC3-core dimer (3, 4, 5, 6, 7, 8, 9) and small regulationary subunits, ssSPTa or ssSPTb (10, 11, 12) and ORMD3 proteins (13, 14).

Alterations of the SPT activity caused by mutations of either SPTLC1 or SPTLC2 gene are linked to neurodegenerative diseases such as hereditary sensory and autonomic neuropathy type I (HSAN1) in human (15, 16, 17, 18). HSAN1-related SPT variants utilize l-alanine (l-Ala) or glycine (Gly) rather than l-Ser as the substrate to produce the corresponding atypical 1-deoxysphingolipids (1-deoxySLs), which lack a critical hydroxy moiety, can be neither converted into a series of sphingoglycolipids nor efficiently degraded *in vivo*, and cause toxicity (19, 20, 21, 22). In human, the elevated levels of 1-deoxySLs due to HSAN1 were pointed as a risk factor for macular telangiectasia type 2 (23). A monogenic form of amyotrophic lateral sclerosis caused by mutations of the SPTLC1 gene was also reported (24, 25). Although patients with type 2 diabetes mellitus have no mutations in the genes encoding the SPT subunits, elevated 1-deoxySL levels of the patients have been reported to be correlated with clinical and metabolic phenotypes such as peripheral neuropathy and impaired wound healing (26, 27, 28, 29, 30, 31). It was also suggested that l-Ala supplementation increased the 1-deoxySL level, while l-Ser supplementation suppressed the formation of 1-deoxySLs in an HSAN1 model mouse or HSAN1 patients. Furthermore, when the SPT activity was modulated by the restriction of dietary l-Ser and Gly, the increased endogenous synthesis of toxic 1-deoxySLs inhibited tumor growth in xenograft models in mice (32). In spite of several lines of clinical evidences, the molecular mechanism by which SPT carrying disease-related mutations produces toxic 1-deoxySLs is still unknown.

Sphingolipid-producing prokaryotes such as *Sphingomonas paucimobillis* or *Sphingobacterium multivorum* contain a water-soluble homodimeric SPT (33, 34). We have focused on bacterial SPTs as a model system for eukaryotic SPTs and carried out mechanistic studies on bacterial SPTs using a series of l-Ser analog and *S*-(2-oxoheptadecyl)-CoA, a nonreactive analog of PalCoA (2, 35, 36, 37). The spectroscopic analyses and site-directed mutagenesis studies on bacterial SPTs showed significant importance of a unique histidine residue located at the *re*-side of PLP in the active site; *i.e.*, the histidine residue anchors l-Ser in a correct orientation to prevent unwanted side reactions and acts as the acid catalyst promoting both the Claisen-type condensation and the decarboxylation at later steps. The crystal structures of bacterial SPTs support the interaction between the histidine residue and the carboxy group of the substrate l-Ser (38, 39). Recently, the structures of the human SPT holocomplex were determined by using cryo-EM at a resolution range 2.6 to 3.8 Å (40, 41). The active site architecture of the human SPT determined by cryo-EM was indicated to be almost the same as that of the bacterial enzymes determined by X-ray crystallography (38, 39, 42, 43, 44).

It has been shown that the *S. paucimobillis* SPT can metabolize l-Ala and Gly, in addition to l-Ser, in the presence of large amounts of these amino acids (22). In order to elucidate the substrate-recognition mechanism of SPT, we focused on the enzyme from *S. multivorum* (the *S. multivorum* SPT), the high-resolution crystal structure complexed with Tris of which we recently determined (45). Although cryo-EM is a powerful tool to study the subunit assembly of the eukaryotic SPT complex, the resolution of the data is not high enough to reveal the precise locations/configurations of the side chains of the amino acid residues. Therefore, bacterial SPTs remain to be important as a model system to elucidate the molecular mechanism of SPT variant–dependent toxic 1-deoxySL production. Here, we examined the reactivity of the *S. multivorum* SPT on various amino acids; the *S. multivorum* SPT could convert some amino acids other than l-Ser into the corresponding sphingoid bases in the presence of PalCoA. Furthermore, we obtained high-quality crystals of the ligand-free form and the binary complexes with a series of amino acids, to determine the structures at less than 2 Å resolutions. Based on these findings, how the active site of the *S. multivorum* SPT accommodates various amino acids, together with structural effects of the binding of a nonproductive amino acid, l-threonine (l-Thr), and those of the disease-related mutations of the human SPT, is discussed.

## Results

### Chemical synthesis of 3-keto form of LCB derived from l-Ala, Gly, or l-homoserine

**Figure 1:**
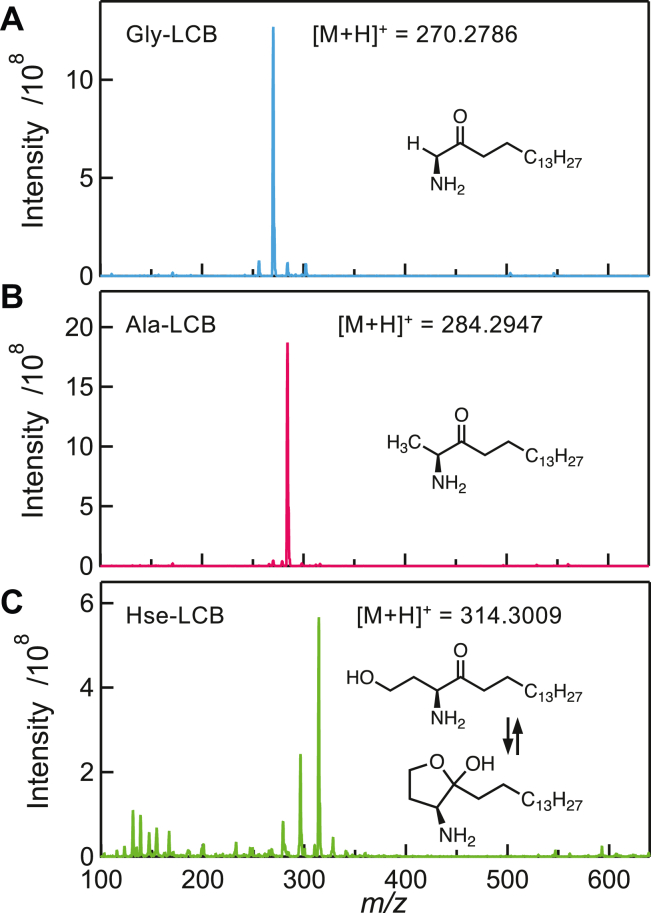
**Synthesis of Gly-, l-Ala–, and l-Hse–derived 3-keto form LCBs.** ESI-MS analysis of Gly-derived LCB (*A*), l-Ala–derived LCB (*B*), and l-Hse–derived LCB (*C*). LCB, long chain base.

In order to assess the activity of LCB synthesis from unnatural substrates by the *S. multivorum* SPT, we synthesized the 3-keto form LCBs derived from Gly, l-Ala, and l-homoserine (l-Hse). The Gly-type and l-Ala-type 3-keto LCBs could be constructed with commercial items, *N*-(*tert*-butoxycarbonyl)–protected amino acids and 1-tetradecanal according to the previously reported procedure (46). Synthesis of the l-Hse-type 3-keto LCB could be achieved by protection of the primary hydroxy group and the amino group of l-Hse and then condensation with 1-tetradecanal. Dozens of milligrams of three kinds of 3-keto LCBs were obtained, and all the 3-keto LCBs synthesized were verified by using ^1^H-, ^13^C-NMR, and ESI-MS (Figs. 1 and S4–S16 in Supporting information). The l-Hse-type 3-keto LCB should reversibly form a cyclized compound, intramolecular hemiketal; the 5-membered ring would be thermodynamically more stable than the linear one. These compounds were used as the authentic standards for TLC analysis of the reaction products.

### Formation of LCBs from unnatural substrates by the *S. multivorum* SPT

**Figure 2:**
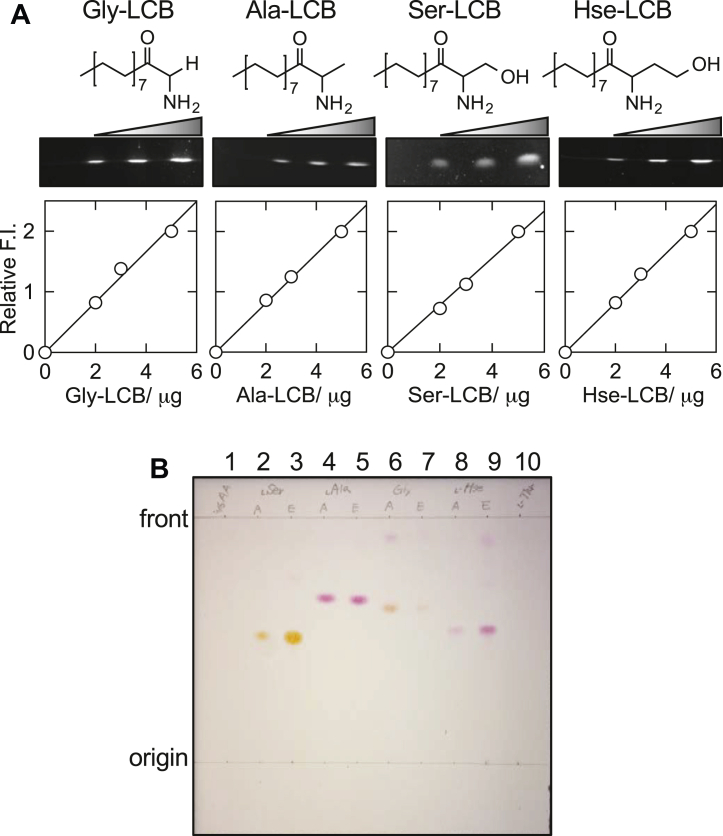
**Formation of LCBs from unnatural substrates by the *Sphingobacterium multivorum* SPT.***A*, chemical structures of Gly-, l-Ala–, l-Ser–, and l-Hse–derived 3-keto form LCBs are shown with their UV-fluorescence images on TLC plates (*middle panels*) and graphs of the fluorescence intensities *versus* the amounts of each LCB after the TLC run (*bottom panels*). *B*, image of the TLC analysis of LCBs visualized with ninhydrin reagent: *lane 1*, extract of the reaction mixture without amino acid substrate; *lane 2*, authentic l-Ser–derived LCB (KDS); *lane 3*, extract of the reaction mixture with l-Ser; *lane* 4, authentic l-Ala–derived LCB; *lane 5*, extract of the reaction mixture with l-Ala; *lane 6*, authentic Gly-derived LCB; *lane 7*, extract of the reaction mixture with Gly; *lane 8*, authentic l-Hse–derived LCB; *lane 9*, extract of the reaction mixture with l-Hse; *lane 10*, extract of the reaction mixture with l-Thr. LCB, long chain base; KDS, 3-ketodihydrosphingosine; SPT, serine palmitoyltransferase.

The synthesized LCBs enabled a significant improvement in both qualitative and quantitative SPT assays. We found that the 3-keto LCBs on TLC plates showed a strong emission upon excitation at 470 nm and that the fluorescence intensity had a linear relationship with the amount of each 3-keto LCB to give a calibration curve (Fig. 2*A*). The range of the quantification of this assay was 2.5 to 50 nmol/spot for each LCB.

We previously reported that the *S*. *paucimobilis* SPT had a broad range of specificity for saturated and unsaturated acyl-CoA substrates of C12 to C22 chain lengths at physiological substrate concentrations (33). The LCB formation from amino acids except for l-Ser has not been detected under physiological conditions. However, when a high concentration (200 mM) of those amino acids and 1 mM of PalCoA were incubated with a large amount (100 μM) of the enzyme, l-Ala, Gly, and l-Hse were converted to corresponding LCBs (Fig. 2*B*). Reaction products were extracted with solvents and separated by TLC. Each reaction product was ninhydrin-positive, the specific color and Rf value of which corresponded to each authentic compound on the TLC plate (Fig. 2*B*). The reaction product was not detected for l-Thr under the conditions examined. Dependency of the reaction rates on amino acid concentrations under the condition of 1 mM PalCoA was examined (Table 1 and Fig. S2). Each LCB product was quantified by the fluorescence intensity based on the standard curve obtained by using the corresponding authentic compound. The values of apparent catalytic efficiency ($k_{cat}^{app}/K_{m}^{app}$) for l-Ala, l-Hse, and Gly were 0.0037, 0.055, and 0.0001 min^–1^ mM^–1^, respectively, and were much smaller than that for l-Ser ($k_{cat}^{app}/K_{m}^{app}$ = 3.6 min^–1^ mM^–1^). Therefore, the LCB production by SPT from amino acids except for l-Ser is considered to be negligible at the physiological substrate concentrations in the bacterial cell.

**Table 1:** Apparent kinetic parameters of the *Sphingobacterium multivorum* SPT

Compounds	$k_{cat}^{app}$^a^ [min^–1^]	$K_{m}^{app}$^a^[mM]	$k_{cat}^{app}/K_{m}^{app}$[min^–1^ mM^–1^]	$K_{d}$^b^[mM]
l-Ser	13 ± 1.0	3.6 ± 0.87	3.6	0.45 ± 0.050
Gly	0.008 ± 0.002	77 ± 8.1	0.0001	71 ± 5.5
l-Ala	0.42 ± 0.011^c^	110 ± 9.0^c^	0.0037	65 ± 10^b^
l-Hse	4.5 ± 0.28	82 ± 14	0.055	3.6 ± 0.20
l-Thr	n.d.^d^	n.d.^d^	n.d.^d^	8.2 ± 1.0
Tris	n.d.^d^	n.d.^d^	n.d.^d^	40 ± 5.6

Data are shown as mean ± SD of three or more measurements.

Abbreviations: PMP, pyridoxamine 5′-phosphate; PLP, pyridoxal-5′-phosphate; SPT, serine palmitoyltransferase.

a The $k_{cat}^{app}$ and $K_{m}^{app}$ values were determined in the presence of 1 mM PalCoA.

b The $K_{d}$ value for l-Ala was determined by measuring the absorption intensity at 424 nm immediately after SPT was mixed with l-Ala.

c The reaction mixture was supplemented with 0.1 mM PLP because the PLP–l-Ala external aldimine was slowly converted to PMP and pyruvate.

d n.d., not determined.

**Figure 3:**
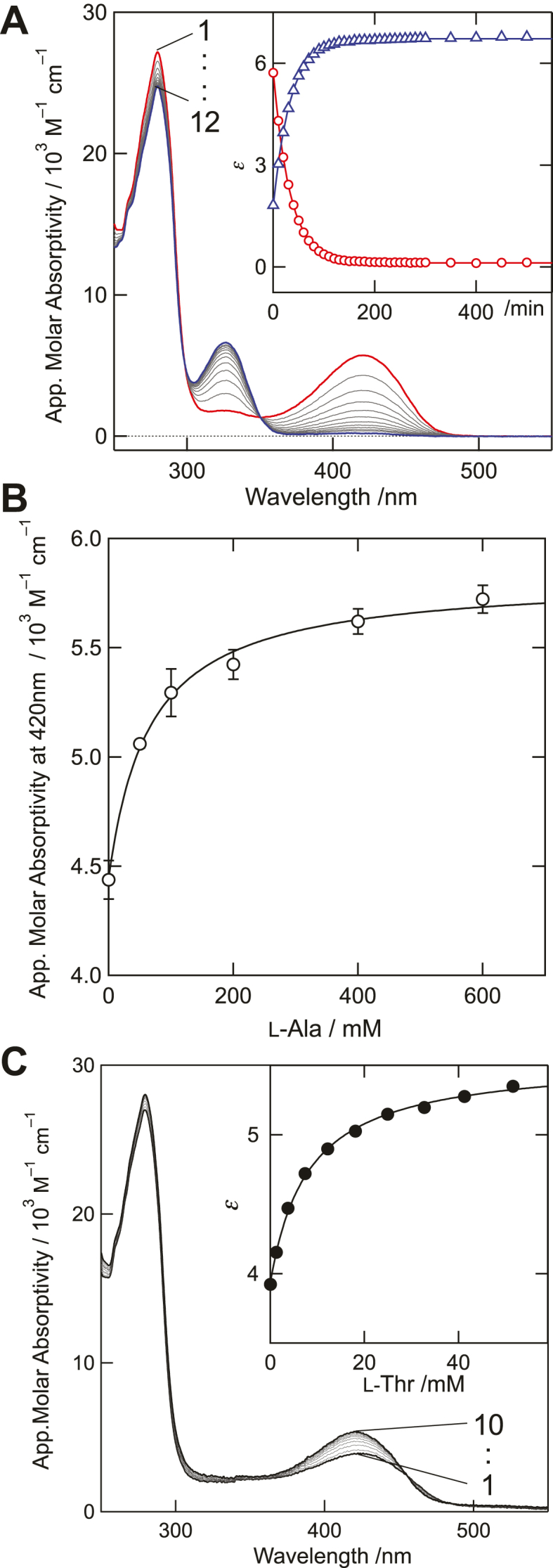
**Spectroscopic analysis of the reaction of the *Sphingobacterium multivorum* SPT with l-Ala or l-Thr.***A*, the UV/vis absorption spectra of SPT (10 μM) in the presence of 800 mM l-Ala were measured at time intervals of 10 min for 500 min. Each line numbered 1 to 12 corresponds to the spectrum at 0, 10, 20, 30, 40, 50, 60, 80, 90, 110, 150, and 500 min, respectively, after SPT was mixed with l-Ala. The inset shows the time courses of changes in the absorption at 420 nm (circle, *red*) and 328 nm (triangle, *blue*). The lines represent theoretical fits to a single-exponential equation ($y=A\times e^{\mathrm{-k}\mathrm{\cdot t}}$), where *k* is 0.029 ± 0.00024 min^–1^. *B*, spectroscopic titration of SPT with l-Ala. Absorbance at 420 nm in the presence of 0, 50, 100, 200, 400, and 600 mM l-Ala at 0 min was measured, and apparent molar absorptivities at 420 nm were plotted against l-Ala concentrations. The data were fit with a theoretical curve with the *K*_d_ value of 65 mM. *C*, absorption spectra of SPT (10 μM) in the presence of 0, 1.3, 3.7, 7.4, 12.2, 18.1, 24.9, 32.7, 41.3, and 51.6 mM l-Thr (lines 1–10, respectively). The inset shows the titration of SPT with l-Thr monitored at 422 nm, fitted with a theoretical curve with the *K*_d_ value of 8.2 mM. SPT, serine palmitoyltransferase.

Next, binding of the amino acids to the *S. multivorum* SPT was examined by spectroscopic titration. When SPT was incubated with l-Ala, PLP and l-Ala were gradually converted to pyridoxamine 5′-phosphate (PMP) and pyruvate with a half-life of τ_1/2_ = 24.0 ± 0.2 min (Fig. 3*A*). Therefore, the absorption at 420 nm just after SPT was mixed with l-Ala was measured and plotted against concentrations of l-Ala (Fig. 3*B*), and the *K*_d_ value for l-Ala was estimated as 65 ± 10 mM (Table 1). For the other amino acids, only spectral changes corresponding to the external aldimine formation between PLP and the amino acids were observed, and side reactions such as PMP formation were not detected. As shown in Table 1, the *K*_d_ values for all the amino acids tested were 10 to 200 times larger than that for l-Ser (0.45 ± 0.050 mM). The nonproductive ligand l-Thr also bound to SPT to form the external aldimine intermediate (Fig. 3*C*). The *K*_d_ value for l-Thr was 8.2 ± 1.0 mM, indicating that the binding affinity for l-Thr is higher than the productive amino acids l-Ala and Gly.

### Crystal structure of the ligand-free form of SPT

**Figure 4:**
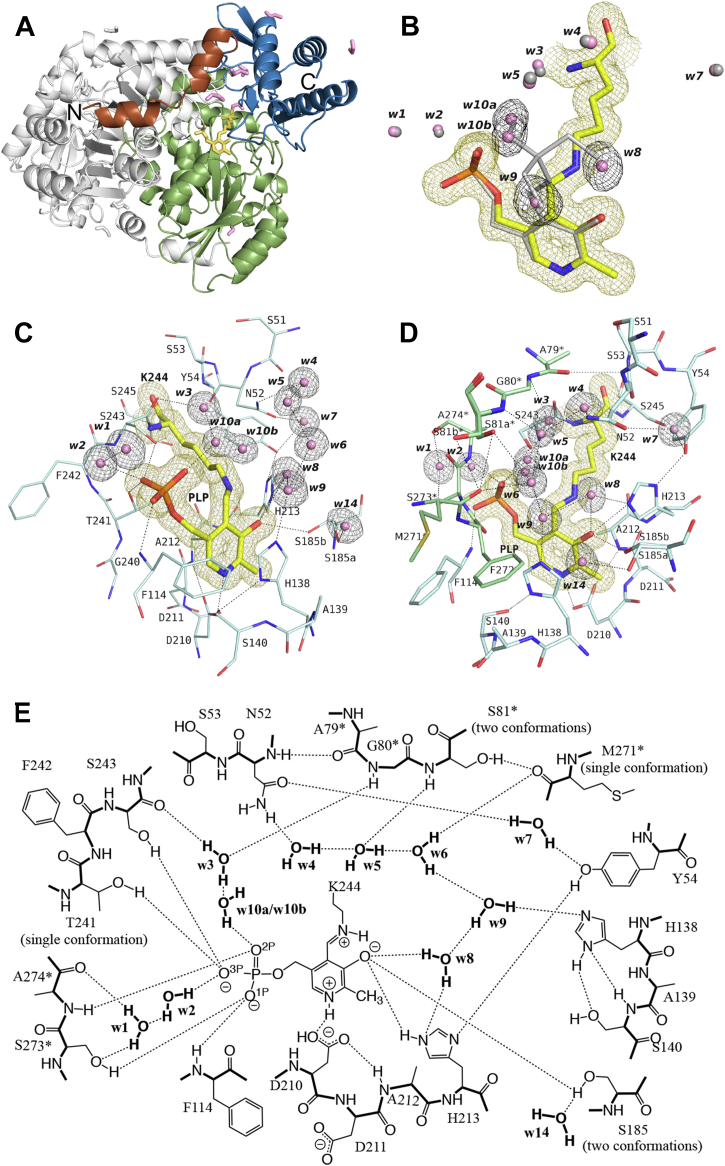
**Crystal structure of the ligand-free form of SPT.***A*, ribbon diagram of the symmetric dimer of the ligand-free form of the *Sphingobacterium multivorum* SPT. SPT crystallized with one molecule per asymmetric unit, and a dimer was generated by crystallographic symmetry. The N- and C-termini of one subunit are labeled N and C. The three structural domains of the subunit are colored to highlight the N-terminal domain (*brown*), the small domain (*blue*), and the large domain (*green*). The PLP–Lys244 internal aldimine and ethylene glycol in the subunit are shown as *yellow*- and *pink*-colored stick models, respectively. Polypeptide, the PLP–Lys244 internal aldimine, and ethylene glycol included in the other subunit are shown in *gray*. *B*, the *gray*-colored model for the PLP–Tris external aldimine and water molecules of the SPT–Tris complex (PDB: 8GUH) was superimposed onto the PLP–Lys244 internal aldimine of the ligand-free SPT structure. *B*–*D*, close-up views of the active site. Calculated 2*F*o–*F*c omit electron density map contoured at 1 σ level is shown for the PLP–Lys244 internal aldimine and water molecules by *yellow* and *black* meshes, respectively. The amino acid residues and the PLP–Lys244 internal aldimine moiety are presented in stick representation. The carbon atoms of the aldimine moiety and the polypeptides of the two subunits are color-coded by *yellow*, *cyan*, and *green*, respectively, and their nitrogen, oxygen, and phosphorus atoms are colored *blue*, *red*, and *orange*, respectively. The water molecules are drawn as spheres. *E*, schematic overview of the interactions among the residues and water molecules in the ligand-free SPT active site. Hydrogen bonds are represented by *black dashed* lines. The N–C–C polypeptide backbones and water molecules are shown in bold. To distinguish residues from the two subunits of a biological dimer, residues from the opposite subunit are labeled with an asterisk. PLP, pyridoxal-5′-phosphate; SPT, serine palmitoyltransferase.

In a previous study, we improved the crystal quality of the *S. multivorum* SPT by optimizing the purification and crystallization procedures (45). The *S. multivorum* SPT could be crystallized only in a Tris-buffered solution, yielding the crystal of the SPT–Tris complex. The crystal of the ligand-free form of SPT was prepared by soaking the preformed crystal of the SPT–Tris complex into a Tricine-buffered Tris-free solution for 90 min before the X-ray diffraction data collection (Table. S1). This Tricine-soaked crystal diffracted to 1.40 Å resolution, and the diffraction data were indexed in the tetragonal space group *P*4_1_2_1_2, with unit-cell parameters *a* = *b* = 61.6 Å, *c* = 208.4 Å, *α* = *β* = *γ* = 90°. A summary of the data statistics is presented in Table 2. The crystal structure was determined at a resolution of 1.40 Å and refined to *R*_work_ and *R*_free_ values of 0.168 and 0.202, respectively, with a Cruickshank diffraction-component precision index (Cruickshank DPI) of 0.0625 Å. The refinement statistics are also summarized in Table 2. The final model contained 394 amino acid residues, one PLP, 535 water molecules, and seven ethylene glycol molecules per protomer (Fig. 4*A*). The model contained 394 of 398 amino acid residues from Ser2 to Val395, and the last three C-terminal residues did not have defined electron density like those of the SPT–Tris complex (45). Judging from the lower Cruickshank DPI of the Tricine-soaked SPT crystal than that of the SPT–Tris complex crystal (0.1067 Å), it is considered that the quality of the SPT crystal was not deteriorated by the Tricine-soaking treatment.

**Table 2:** Data collection and refinement statistics

Parameter	Value(s) for:					
	Ligand-free form	l-Ser complex	l-Hse complex	Gly complex	l-Ala complex	l-Thr complex
Data collection^a^						
Diffraction source	SP8 BL26B2	PF BL17A	Rigaku FR-X	PF BL17A	Rigaku FR-X	PF BL5A
Wavelength (Å)	0.9	0.98	1.5418	0.98	1.5418	0.98
Temperature (K)	100	100	100	100	100	100
Detector	Rayonix MX225HS	Dectris Eiger X16M	Rigaku RAXIS-VII	Dectris Eiger X16M	Rigaku RAXIS-VII	Dectris Pilatus3 S6M
Crystal-detector distance (mm)	100	180	100	180	100	182.2
Rotation range per image (°)	0.1	0.25	0.5	0.25	0.5	0.25
Total rotation range (°)	135	135	135	135	270	135
Exposure time per image (s)	0.1	0.5	60	0.5	60	0.5
Space group	*P*4_1_2_1_2	*P*4_1_2_1_2	*P*4_1_2_1_2	*P*4_1_2_1_2	*P*4_1_2_1_2	*P*4_1_2_1_2
Cell dimensions						
*a*, *b*, *c* (Å)	61.61, 61.61, 208.35	61.28, 61.28, 207.79	61.24, 61.24, 208.10	61.57, 61.57, 207.77	61.23, 61.23, 208.18	61.08, 61.08 207.89
α, β, γ (°)	90, 90, 90	90, 90, 90	90, 90, 90	90, 90, 90	90, 90, 90	90, 90, 90
Resolution range (Å)	50-1.40 (1.48-1.40)	50-1.50 (1.59-1.50)	50-1.55 (1.64-1.55)	50-1.45 (1.54-1.45)	50-1.54 (1.63-1.54)	50-1.45 (1.54-1.45)
⟨ *I*/σ(*I*)	20.6 (2.4)	15.0 (3.9)	13.6 (4.1)	22.8 (3.7)	27.1 (6.7)	31.4 (5.6)
*R*_meas_^b^	0.062 (1.089)	0.084 (0.370)	0.102 (0.261)	0.059 (0.604)	0.072 (0.260)	0.041 (0.316)
*CC*_1/2_^c^	1.00 (0.848)	0.998 (0.949)	0.997 (0.924)	0.999 (0.969)	0.999 (0.965)	1.00 (0.967)
*R*_p.i.m._	0.019 (0.372)	0.028 (0.124)	0.036 (0.117)	0.020 (0.221)	0.017 (0.075)	0.014 (0.106)
Completeness (%)	100.0 (99.9)	99.2 (99.1)	98.3 (92.6)	100.0 (100.0)	98.3 (89.5)	100.0 (96.7)
Redundancy	10.8 (10.7)	9.3 (8.9)	8.3 (5.1)	9.6 (9.7)	17.8 (12.1)	9.3 (8.9)
Mosaicity (°)	0.121	0.190	0.259	0.106	0.216	0.169
Refinement^a^						
No. of reflections, working set	71987 (5274)	57569 (4227)	51776 (3298)	64717 (4654)	52896 (3262)	63757 (4635)
Resolution range (Å)	43.60-1.40 (1.44-1.40)	45.94-1.50 (1.54-1.50)	45.95-1.55 (1.59-1.55)	46.06-1.45 (1.49-1.45)	45.96-1.54 (1.58-1.54)	43.23-1.45 (1.49-1.45)
*R*_work_*/R*_free_^d^	0.168/0.202	0.148/0.184	0.183/0.223	0.188/0.229	0.172/0.216	0.145/0.174
Completeness (%)	100.0	99.3	98.4	100.0	98.4	99.9
Cruickshank DPI (Å)	0.0636	0.0702	0.0939	0.0766	0.0876	0.0605
No. of non-H atoms^e^						
Protein	3223	3336	3202	3197	3245	3238
PLP-external aldimine	24	22	23	20	21	23
Ethylene glycol	28	24	20	32	24	36
Water	566	505	489	415	504	475
R.m.s. deviations^f^						
Bonds (Å)	0.013	0.012	0.011	0.013	0.012	0.013
Angles (°)	1.895	1.829	1.698	1.894	1.769	1.867
Average *B* factors (Å^2^) g						
Protein	21.1	20.0	14.0	20.5	16.4	17.4
PLP-external aldimine	16.8	13.9	8.3	16.4	12.3	11.6
Ethylene glycol	45.2	45.2	34.0	45.4	40.4	38.0
Water	34.6	34.6	26.2	33.3	29.4	33.0
Ramachandran plot						
Most favored (%)	98	97	98	97	98	97
Allowed (%)	2	3	2	3	2	3
PDB ID	8H1W	8H1Q	8H1Y	8H20	8H21	8H29

Abbreviation: PLP, pyridoxal-5′-phosphate.

a One crystal was used for each data set. Values in parentheses are for the highest-resolution shell.

b *R*_mears_ = $\sum_{hkl}{\left[N/\left(N-1\right)\right]}^{1/2}\times \sum_{i}\left|I_{i}\left(hkl\right)-\left\langle I\left(hkl\right)\right\rangle \right|/\sum_{hkl}\sum_{i}I_{i}\left(hkl\right)$ , where $I_{i}\left(hkl\right)$ is the intensity of the *i*th observation of reflection (hkl) and *N* is the redundancy.

c Values of the highest-resolution shells. *CC*_1/2_, Pearson correlation calculated between two random half data sets.

d *R*_free_ was calculated as for *R*_cryst_ but is calculated for 10% of the reflections that were chosen at random and omitted from the refinement process (56).

e Corresponding two atoms of 50% occupancies in the double conformers were counted separately.

f Root mean square deviations.

g The average temperature factor was calculated based on the values of no. of non-H atoms.

In the Tricine-soaked SPT crystal, the electron density of Tris completely disappeared, and the clear electron density of a covalent bond between the ε-amino group of Lys244 and the C4A atom of PLP was observed (Fig. 4*B*). This result shows that the Tris molecule was released from the SPT active site and that PLP regenerated a Schiff-base bond with Lys244, the internal aldimine, in the crystal. Three water molecules, w8, w9, and w10, were assigned at the positions once occupied by the three hydroxymethyl groups of Tris. One of them (w9) interacted with Nε2 of His138 as shown in Figure 4*C*. Another water molecule (w8) interacted with both O3′ of PLP and Nε2 of His213 as shown in Figure 4*D*, and the other one (w10) was split into two electron densities with the occupancy of 0.5 each, one (w10a) of which interacted with the phosphate group of PLP. The torsion angles of C4–C5–C5A–O4P (69.6°) and C5–C5A–O4P–P (−145.6°) of PLP in the ligand-free SPT crystal were close to those of the SPT–Tris complex, 77° and −143°, respectively (Fig. 4*B*). The interactions surrounding the PLP molecule in the SPT active site were basically conserved: the side chain of His138 stacked to the pyridine ring of PLP, and Ala212 interacted with the opposite face of PLP *via* van der Waals interactions. The side chain of Asp210 formed a hydrogen bond/electrostatic interaction with N1 of PLP. The O3′ atom of PLP formed a hydrogen bond to Nε2 of His213, and the phosphate moiety of PLP was anchored by polar interactions with the active site residues, such as the main chain N atoms of Phe114 and Ala274∗ and the side chains of Ser243 and Ser273∗, and hydrogen-bonding networks *via* two water molecules, w1 and w2 (Fig. 4, *C*, *D*, and *E*). (To distinguish the residues from the two subunits of a biological dimer, we labeled the residues from the opposite subunit with an asterisk.) The side chain hydroxy group of Thr241 was directed to the phosphate group of PLP. The side chain of Ser185 took two discrete conformations; the hydroxymethyl group in one conformation is directed to O3′ of PLP and that in the other conformation is directed away from PLP to interact with a water molecule, w14 (Fig. 4, *C* and *D*). The side chain of Ser81∗ was also revealed as having two conformations.

### Crystal structure of the SPT–l-Ser complex

**Figure 5:**
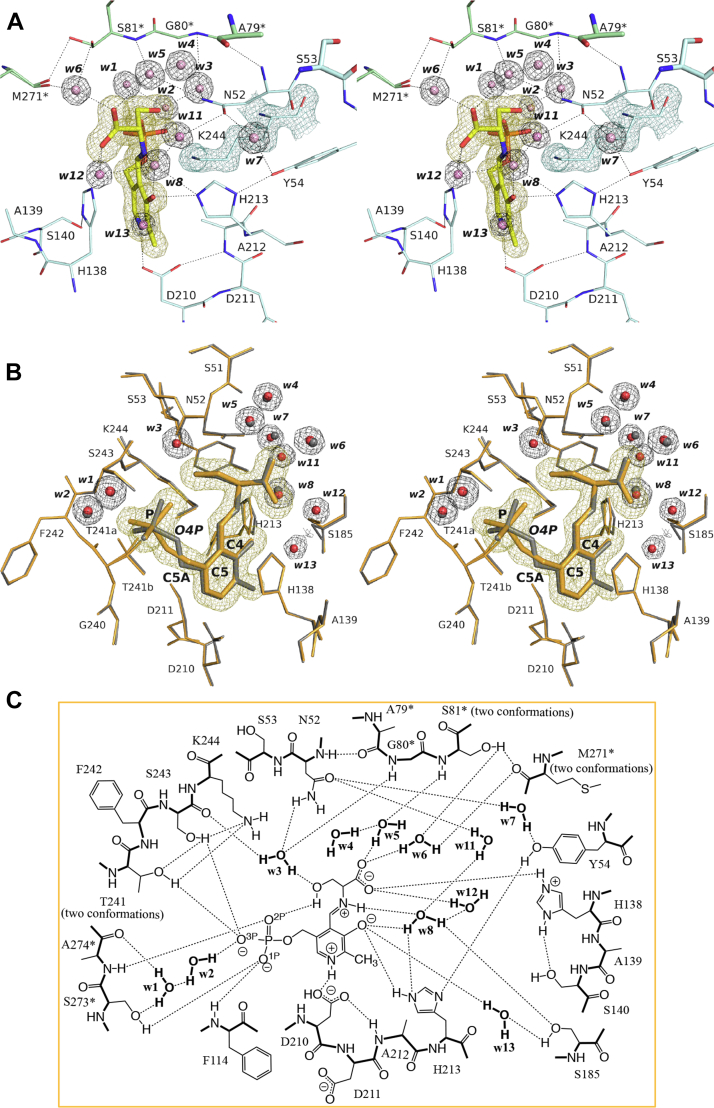
**Crystal structure of the active site of the SPT–l-Ser complex.***A*, stereo representation of a close-up view of the active site. The carbon atoms of the aldimine moiety and the polypeptides of the two subunits are color-coded by *yellow*, *cyan*, and *green*, respectively, and their nitrogen, oxygen, and phosphorus atoms are colored *blue*, *red*, and *orange*, respectively. The water molecules are drawn as *pink* spheres. Calculated 2*F*o–*F*c omit electron density map contoured at 1 σ level is shown for Lys244 (*blue* mesh), the PLP–l-Ser external aldimine (*yellow* mesh), and water molecules (*black* mesh). *B*, stereo representation of the active site viewed from another angle. The improved structure reported in the present study (*orange* and *red*) is superimposed onto the previously reported structure (3A2B; *gray*). Calculated 2*F*o–*F*c omit electron density map contoured at 1 σ level is shown only for the improved structure. *C*, schematic overview of the interactions among the amino acid residues and water molecules in active site of the SPT–l-Ser complex shown in the same manner as in Figure 4*E*. PLP, pyridoxal-5′-phosphate; SPT, serine palmitoyltransferase.

The crystal of the binary complex of SPT with l-Ser could be prepared by soaking the crystal of the SPT–Tris complex into the Tricine-based buffer supplemented with 285 mM l-Ser for 5 min (Table S1). The crystal structure was determined at a resolution of 1.50 Å (Fig. 5*A*). A summary of the data statistics is presented in Table 2. The quality of the SPT–l-Ser complex structure was improved compared to a previously reported structure (PDB: 3A2B) (38). Specifically, the crystallographic *R*_work_/*R*_free_ values of 0.148/0.184 for the present SPT–l-Ser complex structure were smaller than the values of 0.211/0.270 for 3A2B, indicating higher crystallographic reliability. The average B-factor value, 20.0 Å^2^, for all atoms of the present structure was also smaller than that, 28.5 Å^2^, for 3A2B. Consequently, the Cruickshank DPI, 0.0702 Å, was significantly improved compared to 0.39 Å for 3A2B, demonstrating improvement in the coordinate accuracy.

The 2*F*o-*F*c electron density map calculated from the data set clearly indicated the formation of the external aldimine intermediate (Fig. 5, *A* and *B*). A model of the PLP–l-Ser external aldimine could be well assigned at the occupancy of 1.0 to this electron density map. The torsion angles of C4–C5–C5A–O4P (80.5°) and C5–C5A–O4P–P (−145.2°) of PLP in the present SPT–l-Ser complex structure (Fig. 5*B*) were different from those in 3A2B (14.4° and 150.9°) and were close to those of the ligand-free form SPT (69.6° and −145.6°) (Fig. 4*B*). The interactions surrounding PLP were basically the same as those of the ligand-free form SPT (Figs. 4*E* and 5*C*). However, the side chain of Ser185, which took two conformations in the ligand-free form, converged to a single conformation in the SPT–l-Ser complex (Fig. 5, *B* and *C*). The loop region of Ile183–Met186, including Ser185, slightly moved to expand the space of the active site, and the positive peaks of the difference Fourier map of the electron density in this space revealed four water molecules, w8, w11, w12, and w13 (Fig. 5*B*). One water molecule, w8, lay between the side chain of Ser185 and the Schiff base (aldimine) nitrogen, and its B-factor was 20.30 Å^2^. The other three water molecules, w11, w12, and w13, directly interacted with the side chain of Asn52, the carboxy group of l-Ser, and O3′ of PLP, respectively. These three water molecules had additional interactions with water molecules located outside the protein, so that they had relatively large B-factor values of 33.82, 31.53, and 28.34 Å^2^, respectively. The distances between w13 and Oγ of Ser185 and between w11 and w8 were 2.3 Å and 2.4 Å, respectively, too short as hydrogen bonding distances. Therefore, the coordinates of w13 and w11 might be misaligned from the real positions.

The carboxy group of l-Ser formed direct hydrogen bonds to Nε2 of His138 and three water molecules, w5, w6, and w12. And, *via* two of these water molecules, w5 and w6, the l-Ser–derived carboxy group formed water-mediated hydrogen bonds to Ser81∗ (the main-chain amide nitrogen and the side-chain hydroxy group) and Met271∗ (the main-chain carbonyl group). The hydroxy group of l-Ser formed a direct hydrogen bond to the phosphate group of PLP and water-mediated hydrogen bonds to the side-chain of Asn52, the main-chain carbonyl group of Ser243, and the main-chain amide nitrogen of Gly80∗. In addition, Thr241 was split into two conformations; one conformer interacted with the phosphate group of PLP and the side chain of Ser243, while the other conformer was directed to Lys244 to fix its ε-amino group, which was released from the internal aldimine bond.

### Crystal structures of the SPT–l-Hse, SPT–l-Ala, and SPT–Gly complexes

**Figure 6:**
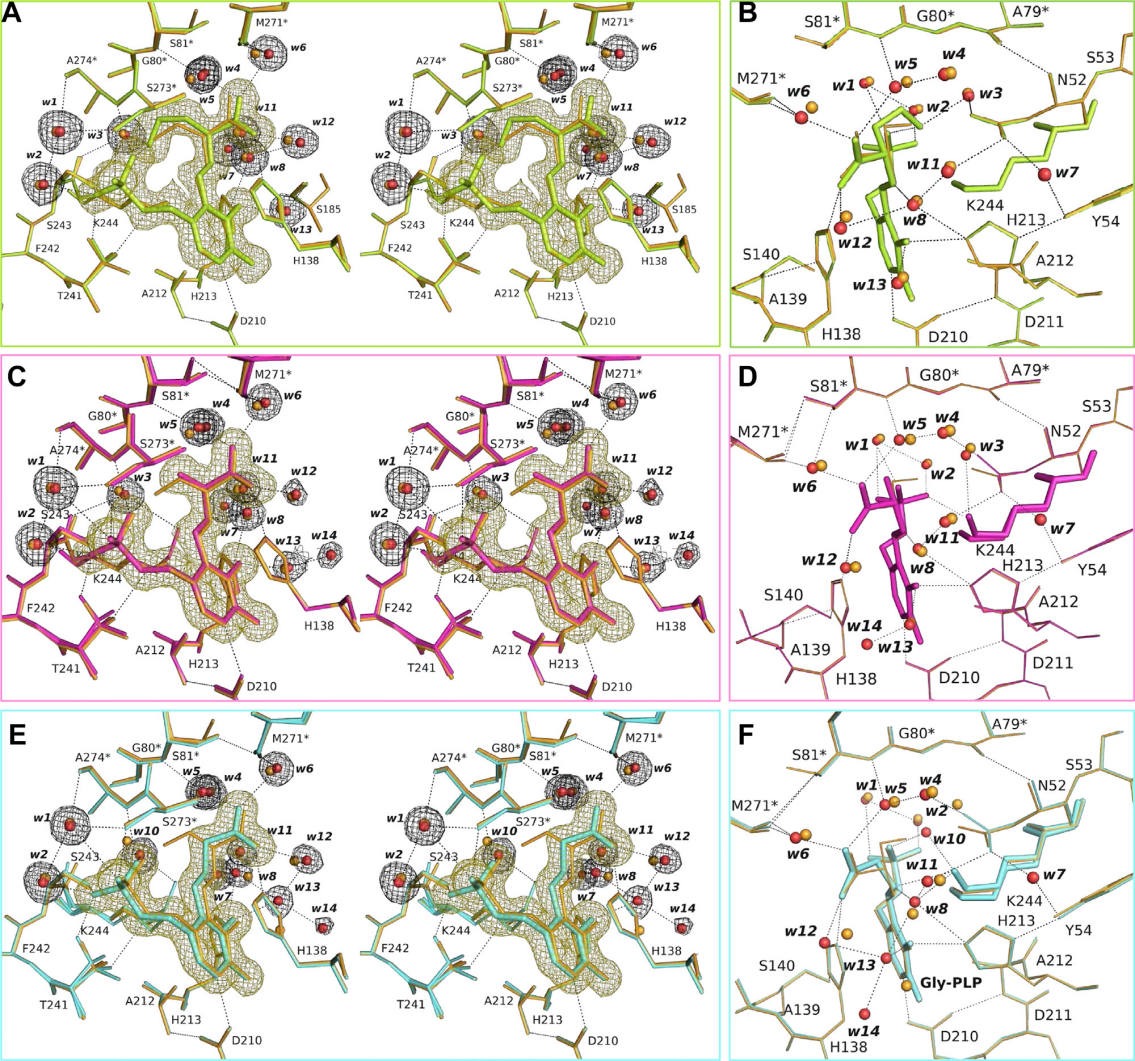
**Crystal structures of the active sites of the SPT–l-Hse, SPT–l-Ala-, and SPT–Gly complexes.***A*, *C*, and *E*, stereo representations of the active sites of SPT complexed with l-Hse (*green* and *red*) (*A*), l-Ala (*pink* and *red*) (*C*), and Gly (*cyan* and *red*) (*E*) superimposed onto the SPT–l-Ser complex (*orange*). Calculated 2*F*o–*F*c omit electron density map contoured at 1 σ level is shown for the PLP–l-Hse, l-Ala, or Gly external aldimine and water molecules by *yellow* and *black* meshes, respectively. *B*, *D*, and *F*, the active sites viewed from another angle for the SPT–l-Hse complex (*green* and *red*) (*B*), the SPT–l-Ala complex (*pink* and *red*) (*D*), and the SPT–Gly complex (*cyan* and *red*) (*F*). The SPT–l-Ser complex (*orange*) is superimposed on to each complex structure. PLP, pyridoxal-5′-phosphate; SPT, serine palmitoyltransferase.

**Figure 7:**
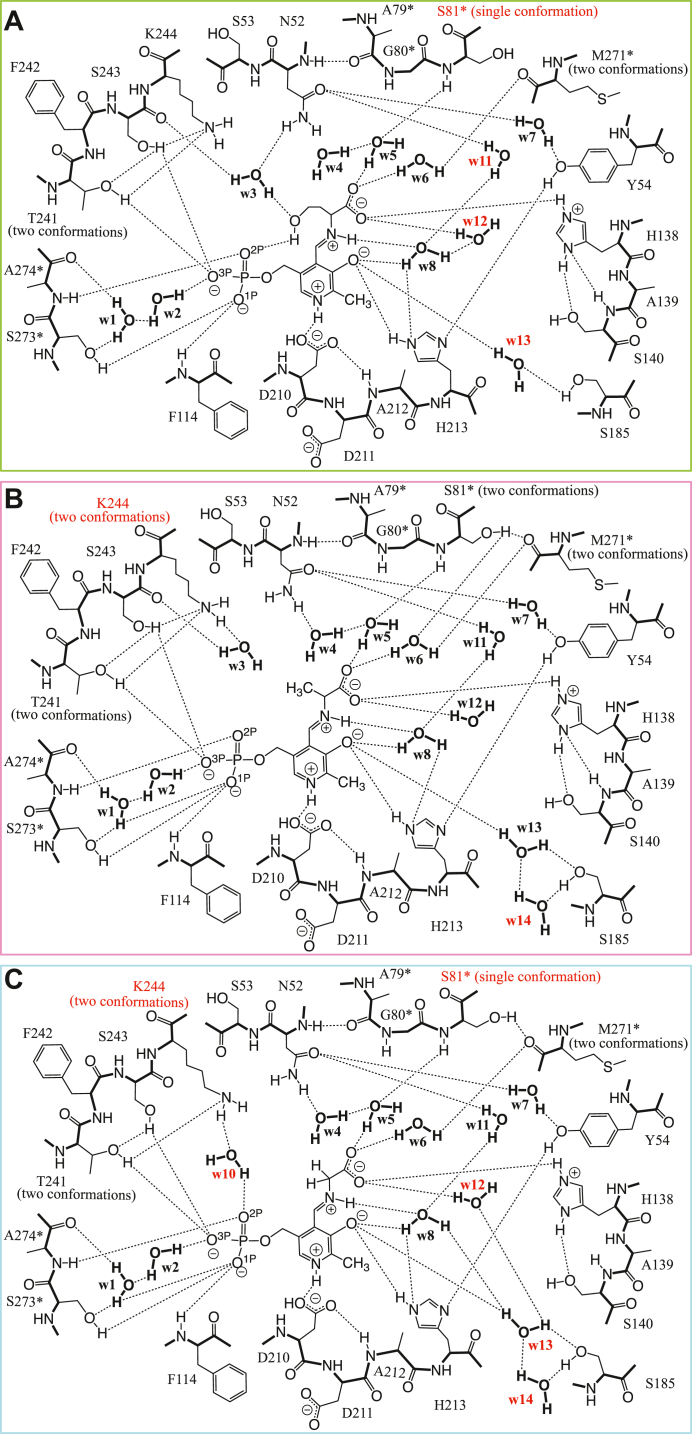
**Schematic overview of the interactions among the amino acid residues and water molecules in the active sites of the SPT–amino acid complexes.** The active site structures of the SPT–l-Hse (*A*), SPT–l-Ala (*B*), and SPT–Gly (*C*) complexes are shown in the same manner as in Figure 4*E*. The amino acid residues and water molecules that were structurally different from those in the SPT–l-Ser complex are shown in *red*. SPT, serine palmitoyltransferase.

The crystals of SPT complexed with the other amino acids could also be prepared by the same soaking procedure (Table S1), and the structure of each binary complex was determined at a high resolution of 1.45 to 1.55 Å. A summary of the data statistics is presented in Table 2. The crystal structure of the SPT–l-Hse complex was determined at a resolution of 1.55 Å and refined to *R*_work_ and *R*_free_ values of 0.183 and 0.223, respectively, with a Cruickshank DPI of 0.0939 Å (Table 2). A clear electron density for the PLP–l-Hse external aldimine was observed (Fig. 6*A*), and the architecture formed by the side chains of the amino acid residues and the water molecules surrounding the PLP–l-Hse external aldimine was basically the same as that observed in the SPT–l-Ser complex (Figs. 6, *A* and *B*, and 7*A*). While Thr241 and Met271∗ existed as two conformers like those in the SPT–l-Ser complex, S81∗ took a single conformation in the SPT–l-Hse complex (Figs. 6*B* and 7*A*). The l-Hse molecule fit into the enzyme active site by bending its 2-hydroxyethyl side chain (–CH_2_-CH_2_-OH). The interactions involving the terminal hydroxy group and the carboxy group of l-Hse were the same as those in the SPT–l-Ser complex. Three water molecules, w11, w12, and w13, had smaller values for the B-factor, 22.12, 26.16, and 21.01 Å^2^, respectively, than those in the l-Ser complex (33.82, 31.53, and 28.34 Å^2^).

The crystal structure of the SPT–l-Ala complex was determined at a resolution of 1.54 Å and refined to *R*_work_ and *R*_free_ values of 0.172 and 0.216, respectively, with a Cruickshank DPI of 0.0876 Å (Table 2). As shown in Figure 2, when l-Ala was added to the *S. multivorum* SPT in solution, the external aldimine formed between PLP and l-Ala was gradually converted to PMP and pyruvate. However, this abortive transamination did not proceed significantly in the SPT crystal soaked in l-Ala for 5 min (Table S1). The covalent bond between PLP and l-Ala of the external aldimine intermediate was identified in a clear electron density (Fig. 6, *C* and *D*). The transamination reaction should proceed much more slowly in crystal under the cryo-condition than in solution at 25 °C. The two conformer structures of Thr241, Ser81∗, and Met271∗ were the same as those in the SPT–l-Ser complex. The space occupied by the hydroxy group of l-Ser in the SPT–l-Ser complex accommodated no extra water molecules. Instead, in one of the two alternative conformers of Lys244, its side chain was shifted to the space to interact with a water molecule, w3, while the ε-amino group of the other conformer remained to interact with the side chain of Thr241.

The crystal structure of the SPT–Gly complex was determined at a resolution of 1.45 Å and refined to *R*_work_ and *R*_free_ values of 0.188 and 0.229, respectively, with a Cruickshank DPI of 0.0766 Å (Table 2). In spite of the large *K*_d_ value for Gly (71 mM), the Tris molecule was completely exchanged for Gly after the crystal was soaked for 30 min in the 266 mM Gly-containing solution. The electron density suitable for the model of the PLP–Gly external aldimine was obtained with the occupancy of 1.0 (Fig. 6, *E* and *F*). The PLP molecule was held in the active site in the same way as in the other binary complexes (Figs. 6, *E* and *F*, and 7*C*). Also, the binding mode of the carboxy group of Gly was not different from that of the other amino acids. The side chain of Ser81∗ was converged to a single conformation as in the case of the SPT–l-Hse complex. A water molecule, w10, which was not observed in the other structures except for the ligand-free form, was observed in the space accommodating the side chains of the amino acid substrates. This water molecule bridges between the ε-amino group of Lys244 and the phosphate group of PLP. A water molecule, w3, which was observed in all the other structures, disappeared in the Gly complex. The positions of two water molecules, w12 and w13, were significantly changed, resulting in the formation of hydrogen bonds between w8 and w13 and between w12 and w13.

### Crystal structure of the SPT–l-Thr complex

**Figure 8:**
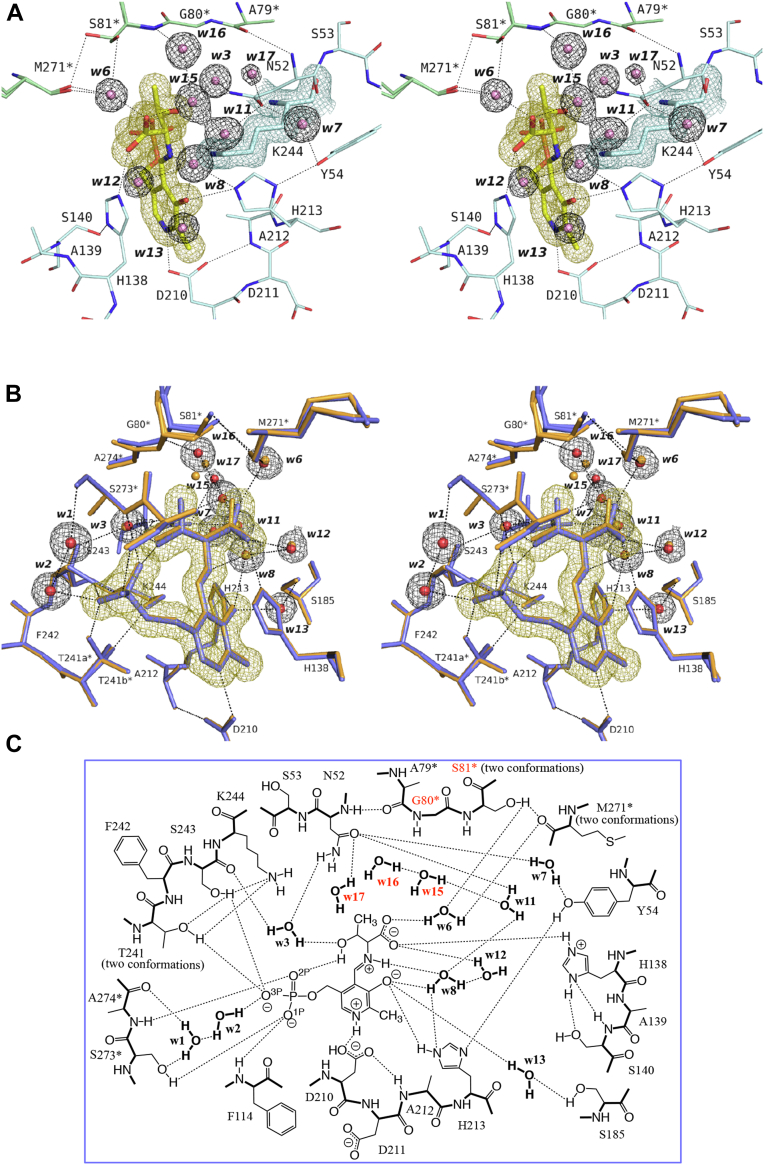
**Crystal structure of the active site of the SPT–l-Thr complex.***A*, stereo representation of the active site. The carbon atoms of the aldimine moiety and the polypeptides of the two subunits are color-coded by *yellow*, *cyan*, and *green*, respectively, and their nitrogen, oxygen, and phosphorus atoms are colored *blue*, *red*, and *orange*, respectively. The water molecules are drawn as *pink* spheres. Calculated 2*F*o–*F*c omit electron density map contoured at 1 σ level is shown for Lys244 (*blue* mesh), the PLP–l-Thr external aldimine (*yellow* mesh), and water molecules (*black* mesh). *B*, stereo representation of the active site viewed from another angle. The structure of the SPT–l-Thr complex (*blue* and *red*) is superimposed onto the SPT–l-Ser complex (*orange*). Calculated 2*F*o–*F*c omit electron density map contoured at 1 σ level is shown only for the SPT–l-Thr complex. *C*, schematic overview of the interactions among the amino acid residues and water molecules in active site of the SPT–l-Thr complex shown in the same manner as in Figure 4*E*. The amino acid residues and water molecules that were structurally different from those in the SPT–l-Ser complex are shown in *red*. PLP, pyridoxal-5′-phosphate; SPT, serine palmitoyltransferase.

The crystal structure of the SPT–l-Thr complex was determined at a resolution of 1.45 Å and refined to *R*_work_ and *R*_free_ values of 0.145 and 0.174, respectively, with a Cruickshank DPI of 0.0656 Å (Table 2). As shown in Figure 2*C*, when l-Thr was added to the SPT solution, changes in the UV/vis spectrum of SPT showed that the transaldimination reaction proceeded to form the PLP–l-Thr external aldimine. Consistent with this result, the experimental data of the electron density and the model of the PLP–l-Thr external aldimine matched very well (Fig. 8*A*). The atoms of the external aldimine model were assigned as the occupancy ratio of 1.0, and the amino acid residues surrounding the PLP moiety in the active site were superimposed onto those of the SPT–l-Ser complex (Fig. 8*B*). As summarized in Figure 8*C*, Thr241, Ser81∗, and Met271∗ were also assigned as having two conformations, and the carboxy group of l-Thr was also fixed by His138 and interacted with two water molecules, w6 and w12. The C4-methyl group of l-Thr pushed and slightly shifted the backbone of Gly80∗ and Ser81∗, leading to larger deviations in the following loop region (see the Discussion section). And, three water molecules, w15, w16, and w17, were newly assigned in the space originally occupied by w4 and w5 (Fig. 8).

## Discussion

In a previous study, we significantly improved the crystal quality of the *S. multivorum* SPT by refining the protein purification and crystallization procedures and utilizing a suitable cryo-protectant, 20% (v/v) ethylene glycol. Soaking the preformed crystal of the SPT–Tris complex into the Tricine-buffered ligand-free or ligand-containing precipitant enabled us to obtain the structures of the ligand-free or binary complexes of SPT without deteriorating the crystal quality. The clear electron density, enabled by high resolution X-ray diffraction data at 1.40 to 1.55 Å resolutions, provided reliable three-dimensional structures of a series of SPT–amino acid binary complexes, which clarified not only the detailed orientations of the side chains of the amino acid residues and the amino acid ligands but also the positions of active-site water molecules.

The architecture surrounding the phosphate group and the pyridine ring of PLP was almost the same among the crystal structures examined in the present study. In each binary complex structure, His138 was stacked parallel to the pyridine ring of PLP and fixed the orientation of the carboxy group of each ligand by a hydrogen bond, and the importance of His138 as a substrate-anchoring site was reaffirmed.

Five amino acid residues in the active site, Ser185, Thr241, Lys244, Ser81∗, and Met271∗, were often determined as two conformers. In the ligand-free form, the side chain of Thr241 took a single conformation to interact with the O^3P^ atom of the phosphate group of PLP. In all the ligand-bound forms, the side chain of Thr241 took two discrete conformations; in one state, it interacted with O^3P^ of PLP, and in the other state, it interacted with the ε-amino group of Lys244, which was released from the internal aldimine bond of the ligand-free form. In the l-Ala and Gly complexes, the side chain of Lys244 was split into two conformations to interact with Oγ of Thr241 or with a water molecule (w3 in the l-Ala complex or w10 in the Gly complex) (Figs. 6 and 7). The small side chains of these amino acid substrates provide some spacial allowance in the active site and might make it possible for Lys244 to take two orientations. As another explanation, because l-Ala and Gly lack a hydroxy group in the side chain and cannot form a hydrogen bond with a water molecule (w3 or w10), this water molecule alternatively forms a hydrogen bond to Lys244, fixing the side chain of Lys244 in a nonpreferred position. These effects on Lys244, a catalytically essential residue, might decrease the binding affinity and the catalytic efficiency of the enzyme (Table1).

**Figure 9:**
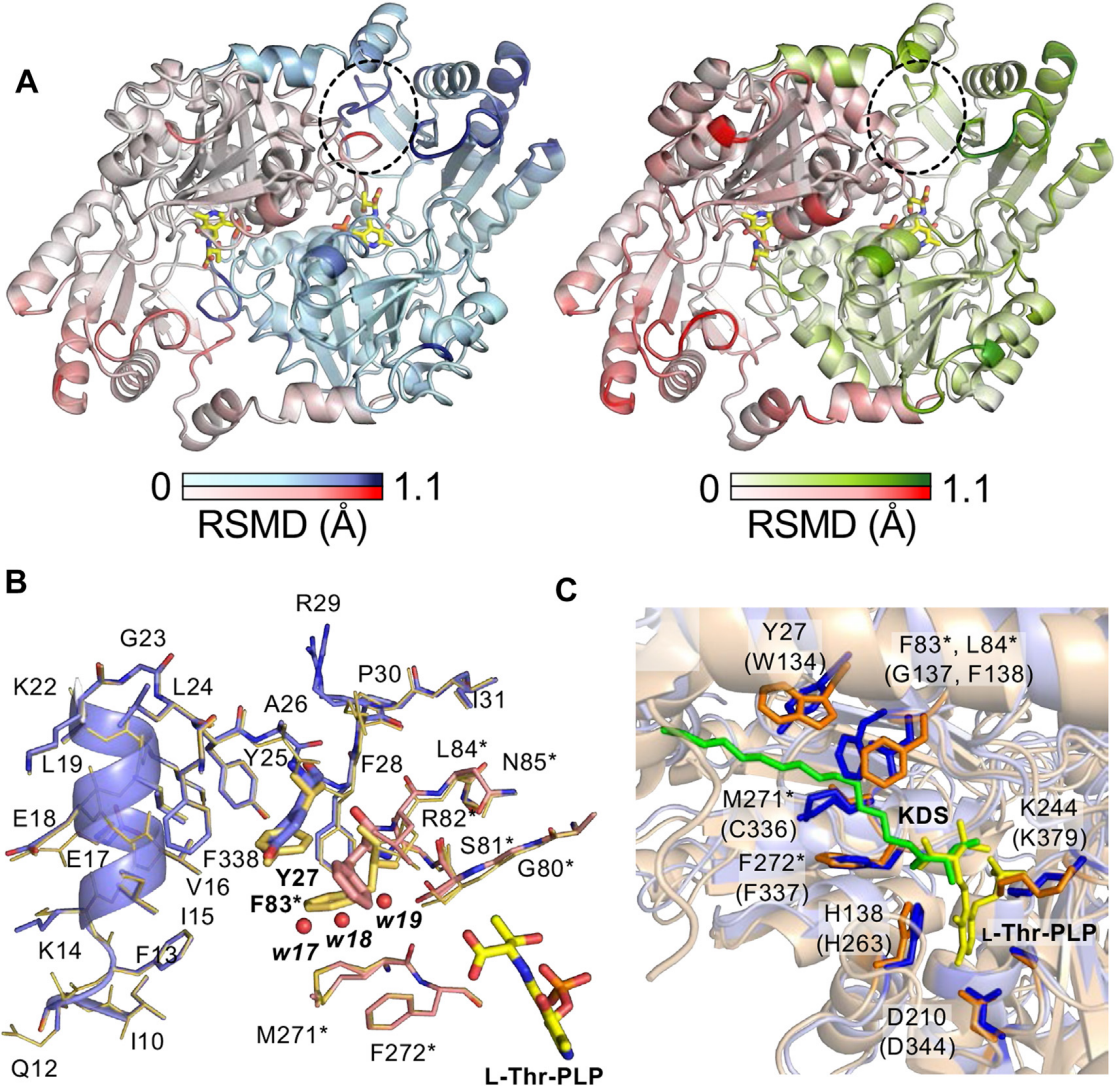
**Structural features of the nonproductive SPT–l-Thr complex.***A*, *left panel*: pairwise structure alignment between the SPT–l-Thr complex and the SPT–l-Ser complex. The structure of the SPT–l-Thr complex is shown by shade of color based on the RMSDs of equivalent atom positions of the protein pair (*white–blue* and *white–red* for the two subunits). Two regions with high RMSDs are indicated by a *dashed* oval. Right panel: pairwise structure alignment between the SPT–l-Hse complex and the SPT–l-Ser complex. The structure of the SPT–l-Hse complex is shown by shade of color based on the RMSDs of equivalent atom positions of the protein pair (*white–green* and *white–red* for the two subunits). The area corresponding to the high RMSD regions of the SPT–l-Thr complex is indicated by a *dashed* oval. *B*, close-up view of the high RMSD regions in the vicinity of the active site of the SPT–l-Thr complex superimposed onto the same regions of the SPT–l-Ser complex. The SPT–l-Thr complex is color-coded by *blue* and *pink* for the polypeptides of the two subunits and *yellow* for the external aldimine moiety. The nitrogen, oxygen, and phosphorus atoms are shown in *blue*, *red*, and *orange*, respectively. The SPT–l-Ser complex is shown by an *orange*-colored thin stick model. *C*, structure alignment between the *Sphingobacterium multivorum* SPT complexed with l-Thr and the human SPT complexed with KDS (PDB: 7k0k). The *S. multivorum* SPT, external aldimine moiety, human SPTLC1/SPTLC2, and KDS are colored *blue*, *yellow*, *orange*, and *green*, respectively. The corresponding amino acid residues of the human enzyme are described in parentheses. KDS, 3-ketodihydrosphingosine; SPT, serine palmitoyltransferase.

Ser185 converged into a single conformation in the ligand-bound forms, while it had two conformations in the ligand-free form. In the ligand-free form, Ser185 was close enough to both O3′ of PLP and a water molecule, w14, to form direct hydrogen bonds, resulting in two alternative conformers (Fig. 4, *C* and *D*). In the ligand-bound forms, a new water molecule, w13, is intercalated between Ser185 and O3′ of PLP, fixing the side chain of Ser185 in one conformation. For the other two residues having two conformations, it is difficult to explain the reason for taking two alternative conformations.

l-Hse (carrying a 2-hydroxyethyl side chain) and l-Thr (carrying a 1-hydroxyethyl side chain) are structural isomers and bind to SPT with the *K*_d_ values of the same order of magnitude (3.6 ± 0.20 mM and 8.2 ± 1.0 mM, respectively). However, l-Thr is a nonproductive ligand, while l-Hse is a substrate of the *S. multivorum* SPT to generate the corresponding LCB product (Fig. 2). To detect and evaluate the difference in the three-dimensional structure between the productive and nonproductive complexes, we performed pairwise structure alignments using the biological assembly (dimer structure) of SPT (Fig. 9). Each complex was compared with the SPT–l-Ser complex, and the aligned structures were shown by shade of color based on the RMSDs of equivalent atom positions of the protein pair. In both structures, amino acid residues with high RMSD values were located outside the catalytic pocket and had disordered side chains exposed to the solvent.

In the pairwise structure alignment between the SPT–l-Thr and SPT–l-Ser complexes, two regions were significantly deviated around the substrate-binding pocket at the dimer interface: a loop region from Gly80∗ to Asn85∗ including a β-turn and a linker region from Ala26 to Pro30 (Fig. 9*A*, left panel). Such deviations were not observed in the alignment between the SPT–l-Hse and SPT–l-Ser complexes (Fig. 9*A*, right panel). The Cα atom of Ser81∗ was pushed by 0.6 Å *via* direct steric repulsion by the Cγ-methyl group of l-Thr. This small shift was propagated to the opposite side of the β-turn and moved the Cα atom of Phe83∗ by 1.12 Å. As shown in Figure 9*B*, these two regions are part of two hydrophobic cores of the two subunits: one comprised of Phe83∗, Leu84∗, Met271∗, and Phe272∗and the other comprised of Phe13, Ile15, Val16, Leu19, Tyr25, Tyr27, Phe28, and Phe338. And these hydrophobic cores come into contact with each other by Phe83∗ and Tyr27. The binding of l-Thr caused large movements of the side chain and main chain of Phe83∗, inducing the shift of the Cα atom of Tyr27 and the rotation of its side chain. The conformational changes of Phe83∗ also generated a space between Phe83∗ and Met271∗, into which three water molecules, w17, w18, and w19, were inserted.

Figure 9*C* shows an overlay drawing of the *S. multivorum* SPT complexed with l-Thr and the human SPTLC1/SPTLC2 dimer, a core dimer, complexed with KDS determined by cryo-EM (7k0k). Tyr27 and Phe83∗ comprise a part of the wall of the binding site for the acyl group of the acyl-CoA substrate or the reaction product. Therefore, conformational changes of the side chains of these residues might inhibit the binding of the acyl-CoA substrate or later steps of the SPT catalysis such as the Claisen-type condensation reaction. It is also possible that, because of the extra Cγ-methyl group of l-Thr, the thioester of PalCoA cannot approach the Cα-position of l-Thr to form a carbon-carbon bond *via* the Claisen-type condensation.

The $K_{m}^{app}$ value for Gly was 21-fold larger than that for l-Ser, but the $k_{cat}^{app}$ value for Gly was 36,000-fold smaller than that for l-Ser. Furthermore, the $K_{m}^{app}$ value for l-Thr was 23-fold larger than that for l-Ser, while the reaction product was not detected for l-Thr. Together with the structural effects of l-Thr binding described above, these results suggest that SPT discriminates l-Ser from other amino acid substrates also in reactions steps following the amino acid binding such as PalCoA binding (Fig. S1, **IIa** → **IIb**), α-deprotonation of the external aldimine (**IIb** → **III**), C–C bond formation (**III** → **IV**), and decarboxylation (**IV** → **V**). Therefore, the crystal structures of a series of SPT–non-l-Ser substrate complexes reported here cannot fully reveal the substrate recognition mechanism of SPT.

**Figure 10:**
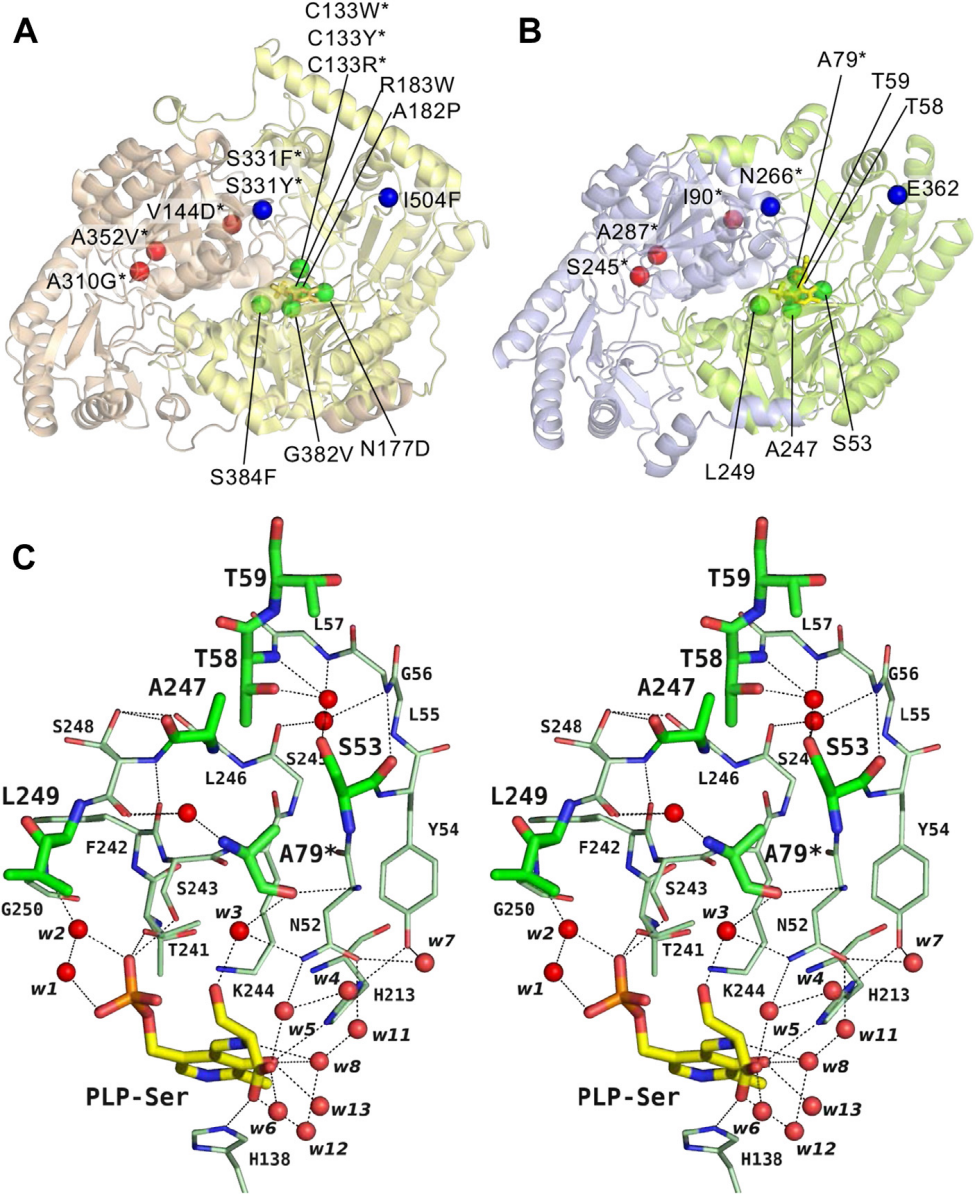
**Projection of disease-related mutation sites on the *Sphingobacterium multivorum* SPT structure.***A*, the Cα atoms of disease-related mutation sites of the human SPTLC1/SPTLC2 dimer (PDB: 7k0k). SPTLC1 and SPTLC2 are colored *wheat* and *yellow*, respectively. *B*, projection of the locations of the Cα atoms of the disease-related residues of the human enzyme on the structure of the *S. multivorum* SPT complexed with l-Ser (PDB: 8H1Q). Two subunits are colored *blue* and *green*. The external aldimine moiety is colored *yellow*. The disease-related amino acid residues were categorized into three groups: near the PLP-binding site (*green*), distributed sporadically on SPTLC1 (*red*), and located on the SPT/ORM protein interface (*blue*). *C*, stereo representation of the active site of the *S. multivorum* SPT–l-Ser complex showing the amino acid residues categorized as ‘near the PLP-binding site’. The carbon atoms of the aldimine moiety and protein subunits are color-coded by *yellow* and *green*, and their nitrogen, oxygen, and phosphorus atoms are colored *blue*, *red*, and *orange*, respectively. The side chain of Ser248 took two conformations. The side chains of several amino acid residues are omitted in this figure for clear representation. PLP, pyridoxal-5′-phosphate; SPT, serine palmitoyltransferase.

We mapped the positions of disease-related missense mutations onto the structure of the human SPTLC1/SPTLC2 dimer complexed with KDS (7k0k) and projected those onto the crystal structure of the *S. multivorum* SPT complexed with l-Ser (8H1Q) (Figs. 10, *A* and *B* and S3). Of the eleven mutations reported in the human SPT, two were located on the SPT/ORM protein interface (*blue*), three were distributed sporadically on SPTLC1 (*red*), and the remaining six were localized near the PLP-binding site (*green*), as previously categorized by Wang *et al.* (40). For all the sites except Ala287∗ of the *S. multivorum* SPT (Ala352 of human SPTLC1), these amino acid residues were not conserved between them. Figure 10*C* is a close up view of the projected mutation sites at the subunit interface of the *S. multivorum* SPT. Ala79∗, corresponding to Cys133 of human SPTLC1, interacts with the side chain of the amino acid substrate *via* the water molecule w3. And Ser81∗, not shown in Figure 10*C* but the second residue from Ala79∗, interacts with the substrate carboxy group *via* the water molecules w5 and w6 (Fig. 5). Ser53, corresponding to Asn177 of human SPTLC2, is located between Asn52 and Tyr54, which participate in hydrogen-bonding networks surrounding the PLP–l-Ser moiety in the active site. Ala247 and Leu249, corresponding to Gly382 and Ser384 of human SPTLC2, are located in the loop structure following the SPT-characteristic PLP-binding motif (-G-T-F-S-K-S-), which contains the essential catalytic residue Lys244. And Gly250, next to Leu249, interacts with the phosphate group of PLP *via* water molecules, w1 and w2. Thr58 and Thr59, corresponding to Ala182 and Arg183 of human SPTLC2, are located far from PLP but in the loop structure following Tyr54, and Thr58 interacts with Ser53 *via* a water molecule. Most of the disease-related mutations of human SPTLC1/SPTLC2 are the replacements of small side chains of the amino acid residues with relatively large ones. Insertion of a bulkier amino acid residue would cause some spacial rearrangements of the side chains of surrounding amino acid residues and water molecules in the active site and affect the intricate hydrogen-bonding networks revealed in the present study, which might influence the substrate recognition of the enzyme to increase the utilization of l-Ala or Gly.

We found that the *S. multivorum* SPT could form the LCBs not only from l-Ala and Gly but also from l-Hse. It was unexpected that SPT had higher affinity for l-Hse than l-Ala and Gly and could metabolize l-Hse more efficiently than them. SPT is unique in showing reactivity towards various amino acid substrates, in contrast to other members of the α-oxamine synthase family, such as 5-aminolevurinate synthase, 8-amino-7-oxononanoate synthase, and 2-amino-3-oxobutyrate CoA ligase, which show very strict substrate specificities. The high-resolution crystal structures of the *S. multivorum* SPT in the present study showed that SPT accommodated a series of amino acids by subtle rearrangements of the side chains of amino acid residues and water molecules in the active site. And none of the disease-related mutations of the human SPT were located within a distance of direct interaction with the bound substrate. Mutations of amino acid residues that directly interact with the substrate might significantly impair the enzyme activity rather than affect the substrate preference. It was previously reported that the substrate specificity of an enzyme was drastically changed by mutations of non-active-site amino acid residues, none of which made direct contact with the bound substrate (47, 48). Future functional and structural studies, in which those disease-related mutations are introduced into the corresponding sites of the *S. multivorum* SPT, might explain the mechanism of how non-active-site mutations affect the substrate specificity of SPT.

The LCB derived from l-Hse could be an interesting precursor of unnatural sphingolipids, where any kind of modifications can be introduced at the head group which is located a little above the lipid bilayer membrane. Bacterial SPTs, especially those with engineered substrate specificities, have the potential as useful catalysts to synthesize other LCBs with interesting biological properties. And further crystallographic studies of SPT, such as a ternary complex consisting of SPT–amino acid–acyl-CoA analog and a binary complex consisting of SPT and product LCBs, would be useful to elucidate the mechanism of ligand binding and catalysis of SPT.

## Experimental procedures

### Expression and purification of the *S. multivorum* SPT

Expression and purification of *the S. multivorum* SPT were performed as previously reported (45). Briefly, *Escherichia coli* BL21 (DE3) pLysS cells (Novagen) harboring the plasmid that express the full length of *S. multivorum* SPT (34) were grown at 37 °C to A_600_ 0.6 to 0.8. SPT expression was induced by adding 0.1 mM IPTG for 4 h. The *E. coli* culture was harvested, and the cells were resuspended in the buffer containing 50 mM Tris–HCl buffer, pH 7.5, and 0.1 mM EDTA and disrupted by sonication. The cell lysate was centrifuged at 18,000*g* for 20 min at 4 °C to obtain the crude extract. The *S. multivorum* SPT was purified by three steps of column chromatography of HiPrepDEAE-FFcrude 16/10, HiPrepButyl-FFcrude 16/10, and the second HiPrepDEAE-FFcrude 16/10 by using an ÄKTA FPLC system (GE Healthcare), and the pooled SPT fractions were desalted using a HiPrepDesalting26/10 column (GE Healthcare) equilibrated with the 20 mM potassium phosphate buffer, pH 7.4, containing 0.1 mM EDTA. Purified SPT was concentrated to 20 mg/ml and stored at 4 °C. The concentration of the purified SPT subunit in solution was spectrophotometrically determined with the molar extinction coefficient of 26,780 M^–1^ cm^–1^ at 280 nm for the PLP form of the enzyme, which was calculated on the basis of the number of tryptophan and tyrosine residues in SPT (34).

### Synthesis of authentic standards of Gly-, l-Ala-, and l-Hse-type 3-keto LCBs

Chemical synthesis of 1-aminoheptadecan-2-one (Gly-type 3-keto LCB), (*S*)-2-aminooctadecan-3-one (l-Ala-type 3-keto LCB), and (*S*)-3-amino-1-hydroxynonadecan-4-one (l-Hse-type 3-keto LCB) was done using commercially available materials based on the method previously reported (46). For details of the synthesis, see Supporting information.

### TLC analysis of LCBs

The assay was performed as follows; 200 mM of amino acid (glycine, l-Ala, l-Ser, l-Hse, or l-Thr) was incubated with 1 mM PalCoA and 100 μM SPT in 100 μl of 100 mM potassium phosphate, pH 7.5, for 60 min at 37 °C. The reactions were terminated by addition of the equal volume of 2 N ammonia. Lipids were extracted by successive addition and mixing of 750 μl of chloroform/methanol (1:2, v/v), 250 μl of chloroform, and 250 μl of 1% KCl. Phases were separated by centrifugation, and the organic phase was recovered. The aqueous phase was reextracted with 200 μl of chloroform, and, after centrifugation, the organic phase was combined with the organic phase from the first extraction. The organic phase was washed with water/chloroform/methanol (47:48:3, v/v), dried, and suspended in chloroform/methanol (2:1, v/v). The extracted lipids were resolved by normal-phase TLC on Silica Gel 60 high-performance TLC plates (Merck) with chloroform/methanol/2 N ammonia (40:10:1, v/v) or chloroform/methanol/triethanol amine (95:5:10, v/v) and visualized by spraying with ninhydrin reagent followed by gentle heating. For the quantitative analysis, the UV-fluorescence intensities of the reaction products on TLC plates were visualized by a Fusion chemiluminescence imaging system using a UV-light box and analyzed by a FUSION Capt software (VILBER; https://www.garvan.org.au/research/capabilities/molecular-genetics/ documents/fusion_manual_2016.pdf).

### Kinetic analysis of SPT

Steady-state kinetic parameters were determined by varying the concentrations of the amino acid substrate in the presence of 1 mM PalCoA in 100 μl of 100 mM potassium phosphate, pH 7.5, at 37 °C. The concentration of SPT and the incubation time were 1 μM and 10 min for l-Ser; 5 μM and 10 min for l-Hse; 20 μM and 30 min for l-Ala; and 100 μM and 120 min for Gly. The range of concentrations of each amino acid substrate was shown in Fig. S2. The reaction termination, lipid extraction, TLC analysis, and quantification of the reaction products on TLC plates were described above. The apparent velocities (min^–1^) *versus* amino acid concentrations (mM) plots were fitted to the Michaelis–Menten equation, v = *V*_max_[S]/(*K*_m_+[S]), by nonlinear regression using the Igor Pro 6.37 (https://www.wavemetrics.com/forum/news-and-announcements/igor-pro-637-released). software (Wave Matrix Inc.) to determine the apparent kinetic parameters, $k_{cat}^{app}$ and $K_{m}^{app}$, of SPT for each amino acid substrate.

### Determination of dissociation constants (K_d_) for amino acids

The titration assay was carried out by using 10 μM SPT in 100 mM potassium phosphate and 0.1 mM EDTA, pH 7.5, at 25 °C. The changes of the 422 nm absorption intensities of SPT upon addition of amino acids were plotted against the final concentrations of the amino acids, and the *K*_d_ values were calculated by fitting to a hyperbolic saturation curve using Igor Pro 6.37. software. UV/vis spectra of SPT were recorded with a Hitachi U-3310 spectrophotometer.

### Crystallization

SPT was crystallized by the sitting drop vapor diffusion method in 24-well plates at 20 °C as described previously (45). Briefly, an aliquot of 2 μl of 20.0 mg/ml protein solution was mixed with 4 μl of the reservoir solution containing 100 mM Tris–HCl (pH 8.5), 200 mM sodium acetate, and 19 to 24 % (w/v) PEG4000. The drop was equilibrated against 500 μl of the reservoir solution for 2 days, and, then, the microbridge was transferred to a new 24-well plate, where the fresh reservoir solution contained 100 mM Tris–HCl (pH 8.5), 200 mM sodium acetate, and 13 to 18 % (w/v) PEG4000. Plate-shaped or cube-shaped crystals were reproducibly grown within 2 to 5 days.

### Crystal soaking

The crystal of the ligand-free form SPT was prepared by soaking the crystal obtained above into the Tris-free solution containing 100 mM Tricine-NaOH (pH 8.5), 200 mM sodium acetate, and 15 % (w/v) PEG4000 for 90 min before data collection. To obtain the crystals of the binary complexes with a series of amino acids, the crystals formed in the presence of Tris were soaked into the Tris-free solution containing 172 to 285 mM amino acids for 5 min (40 min for Gly) before data collection as summarized in Table S1.

### Data collection and structural determination

Crystals were cryo-protected by quick transfer through the soaking solution supplemented with 20% (v/v) ethylene glycol, then flash-frozen in liquid nitrogen or under a N_2_ gas cryostream (100 K). X-ray diffraction data were collected at the BL5A and BL17A beamlines at KEK Photon Factory, λ = 0.98 Å, and the BL26B2 beamline at SPring-8, λ = 0.9 Å. In-house data sets were collected on a Rigaku FR-X rotating anode X-ray source with CuKα radiation (λ = 1.54 Å) equipped with a Rigaku R-AXIS VII image plate as the detector (Rigaku Corporation).

All data were processed and scaled using XDS (49). Initial phases for each structure were determined by the molecular replacement method using MolRep software (https://www.ccp4.ac.uk/html/molrep.html) (50) in the CCP4 program suite (51) using PDB entry 3A2B as the search model after the removal of all water molecules. The model was refined using REFMAC5 (52) in the CCP4 suite, and manual adjustment and rebuilding of the model were performed using the program Coot (53). The quality of the structure was determined by MolProbity (54). Refinement statistics are summarized in Table 2. The atomic coordinates and crystal structures reported in the present study have been deposited in the Protein Data Bank with accession codes 8H1W, 8H1Q, 8H1Y, 8H20, 8H21, and 8H29.

### Structural analysis and generation of figures

Illustrations of the structures and sequence-independent structural superposition were performed using the PyMOL molecular graphics system (DeLano Scientific; http://www.pymol.org).

Sequence alignment analysis and illustration were performed using Genetyx-Mac software v.21.2.1 (Genetyx Corporation) and ESPript 3.0 (55) (https://espript.ibcp.fr).

## Data availability

All data are contained in the article and supporting information.

## Supporting Information

This article contains Supporting information (46, 55).

## Conflict of interest

The authors declare that they have no conflicts of interest with the contents of this article.

## References

[1] Hanada K. (2003). Serine palmitoyltransferase, a key enzyme of sphingolipid metabolism. Biochim. Biophys. Acta.

[2] Ikushiro H., Hayashi H. (2011). Mechanistic enzymology of serine palmitoyltransferase. Biochim. Biophys. Acta.

[3] Buede R., Rinker-Schaffer C., Pinto W.J., Lester R.L., Dickson R.C. (1991). Cloning and characterization of LCB1, a Saccharomyces gene required for biosynthesis of the long-chain base component of sphingolipids. J. Bacteriol..

[4] Nagiec M.M., Baltisberger J.A., Wells G.B., Lester R.L., Dickson R.C. (1994). The LCB2 gene of Saccharomyces and the related LCB1 gene encode subunits of serine palmitoyltransferase, the initial enzyme in sphingolipid synthesis. Proc. Natl. Acad. Sci. U. S. A..

[5] Nagiec M.M., Lester R.L., Dickson R.C. (1996). Sphingolipid synthesis: Identification and characterization of mammalian cDNAs encoding the Lcb2 subunit of serine palmitoyltransferase. Gene.

[6] Hanada K., Hara T., Nishijima M., Kuge O., Dickson R.C., Nagiec M.M. (1997). A mammalian homolog of the yeast LCB1 encodes a component of serine palmitoyltransferase, the enzyme catalyzing the first step in sphingolipid synthesis. J. Biol. Chem..

[7] Hanada K., Hara T., Nishijima M. (2000). Purification of the serine palmitoyltransferase complex responsible for sphingoid base synthesis by using affinity peptide chromatography techniques. J. Biol. Chem..

[8] Hornemann T., Richard S., Rutti M.F., Wei Y., von Eckardstein A. (2006). Cloning and initial characterization of a new subunit for mammalian serine-palmitoyltransferase. J. Biol. Chem..

[9] Hornemann T., Wei Y., von Eckardstein A. (2007). Is the mammalian serine palmitoyltransferase a high-molecular-mass complex?. Biochem. J..

[10] Gable K., Slife H., Bacikova D., Monaghan E., Dunn T.M. (2000). Tsc3p is an 80-amino acid protein associated with serine palmitoyltransferase and required for optimal enzyme activity. J. Biol. Chem..

[11] Han G., Gupta S.D., Gable K., Niranjanakumari S., Moitra P., Eichler F. (2009). Identification of small subunits of mammalian serine palmitoyltransferase that confer distinct acyl-CoA substrate specificities. Proc. Natl. Acad. Sci. U. S. A..

[12] Parthibane V., Lin J., Acharya D., Abimannan T., Srideshikan S.M., Klarmann K. (2021). SSSPTA is essential for serine palmitoyltransferase function during development and hematopoiesis. J. Biol. Chem..

[13] Breslow D.K., Collins S.R., Bodenmiller B., Aebersold R., Simons K., Shevchenko A. (2010). Orm family proteins mediate sphingolipid homeostasis. Nature.

[14] Chauhan N., Han G., Somashekarappa N., Gable K., Dunn T., Kohlwein S.D. (2016). Regulation of sphingolipid biosynthesis by the morphogenesis checkpoint kinase Swe1. J. Biol. Chem..

[15] Bejaoui K., Wu C., Scheffler M.D., Haan G., Ashby P., Wu L. (2001). SPTLC1 is mutated in hereditary sensory neuropathy, type 1. Nat. Genet..

[16] Bejaoui K., Uchida Y., Yasuda S., Ho M., Nishijima M., Brown R.H. (2002). Hereditary sensory neuropathy type 1 mutations confer dominant negative effects on serine palmitoyltransferase, critical for sphingolipid synthesis. J. Clin. Invest..

[17] Hornemann T., Penno A., Richard S., Nicholson G., van Dijk F.S., Rotthier A. (2009). A systematic comparison of all mutations in hereditary sensory neuropathy type I (HSAN I) reveals that the G387A mutation is not disease associated. Neurogenetics.

[18] Rotthier A., Auer-Grumbach M., Janssens K., Baets J., Penno A., Almeida-Souza L. (2010). Mutations in the SPTLC2 subunit of serine palmitoyltransferase cause hereditary sensory and autonomic neuropathy type I. Am. J. Hum. Genet..

[19] Penno A., Reilly M.M., Houlden H., Laura M., Rentsch K., Niederkofler V. (2010). Hereditary sensory neuropathy type 1 is caused by the accumulation of two neurotoxic sphingolipids. J. Biol. Chem..

[20] Ernst D., Murphy S.M., Sathiyanadan K., Wei Y., Othman A., Laura M. (2015). Novel HSAN1 mutation in serine palmitoyltransferase resides at a putative phosphorylation site that is involved in regulating substrate specificity. Neuromol. Med..

[21] Bode H., Bourquin F., Suriyanarayanan S., Wei Y., Alecu I., Othman A. (2016). HSAN1 mutations in serine palmitoyltransferase reveal a close structure-function-phenotype relationship. Hum. Mol. Genet..

[22] Gable K., Gupta S.D., Han G., Niranjanakumari S., Harmon J.M., Dunn T.M. (2010). A disease-causing mutation in the active site of serine palmitoyltransferase causes catalytic promiscuity. J. Biol. Chem..

[23] Gantner M.L., Eade K., Wallace M., Handzlik M.K., Fallon R., Trombley J. (2019). Serine and lipid metabolism in macular disease and peripheral neuropathy. N. Engl. J. Med..

[24] Johnson J.O., Chia R., Miller D.E., Li R., Kumaran R., Abramzon Y. (2021). Association of variants in the SPTLC1 gene with juvenile amyotrophic lateral sclerosis. JAMA Neurol..

[25] Mohassel P., Donkervoort S., Lone M.A., Nalls M., Gable K., Gupta S.D. (2021). Childhood amyotrophic lateral sclerosis caused by excess sphingolipid synthesis. Nat. Med..

[26] Bertea M., Rutti M.F., Othman A., Marti-Jaun J., Hersberger M., von Eckardstein A. (2010). Deoxysphingoid bases as plasma markers in diabetes mellitus. Lipids Health Dis..

[27] Othman A., Rutti M.F., Ernst D., Saely C.H., Rein P., Drexel H. (2012). Plasma deoxysphingolipids: a novel class of biomarkers for the metabolic syndrome?. Diabetologia.

[28] Othman A., Saely C.H., Muendlein A., Vonbank A., Drexel H., von Eckardstein A. (2015). Plasma 1-deoxysphingolipids are predictive biomarkers for type 2 diabetes mellitus. BMJ Open Diabetes Res. Care.

[29] Dohrn M.F., Othman A., Hirshman S.K., Bode H., Alecu I., Fahndrich E. (2015). Elevation of plasma 1-deoxy-sphingolipids in type 2 diabetes mellitus: a susceptibility to neuropathy?. Eur. J. Neurol..

[30] Wilson E.R., Kugathasan U., Abramov A.Y., Clark A.J., Bennett D.L.H., Reilly M.M. (2018). Hereditary sensory neuropathy type 1-associated deoxysphingolipids cause neurotoxicity, acute calcium handling abnormalities and mitochondrial dysfunction *in vitro*. Neurobiol. Dis..

[31] Karsai G., Steiner R., Kaech A., Lone M.A., von Eckardstein A., Hornemann T. (2021). Metabolism of HSAN1- and T2DM-associated 1-deoxy-sphingolipids inhibits the migration of fibroblasts. J. Lipid Res..

[32] Muthusamy T., Cordes T., Handzlik M.K., You L., Lim E.W., Gengatharan J. (2020). Serine restriction alters sphingolipid diversity to constrain tumour growth. Nature.

[33] Ikushiro H., Hayashi H., Kagamiyama H. (2001). A water-soluble homodimeric serine palmitoyltransferase from Sphingomonas paucimobilis EY2395T strain. Purification, characterization, cloning, and overproduction. J. Biol. Chem..

[34] Ikushiro H., Islam M.M., Tojo H., Hayashi H. (2007). Molecular characterization of membrane-associated soluble serine palmitoyltransferases from Sphingobacterium multivorum and Bdellovibrio stolpii. J. Bacteriol..

[35] Ikushiro H., Hayashi H., Kagamiyama H. (2004). Reactions of serine palmitoyltransferase with serine and molecular mechanisms of the actions of serine derivatives as inhibitors. Biochemistry.

[36] Ikushiro H., Fujii S., Shiraiwa Y., Hayashi H. (2008). Acceleration of the substrate Calpha deprotonation by an analogue of the second substrate palmitoyl-CoA in Serine Palmitoyltransferase. J. Biol. Chem..

[37] Shiraiwa Y., Ikushiro H., Hayashi H. (2009). Multifunctional role of His159in the catalytic reaction of serine palmitoyltransferase. J. Biol. Chem..

[38] Ikushiro H., Islam M.M., Okamoto A., Hoseki J., Murakawa T., Fujii S. (2009). Structural insights into the enzymatic mechanism of serine palmitoyltransferase from Sphingobacterium multivorum. J. Biochem..

[39] Raman M.C., Johnson K.A., Yard B.A., Lowther J., Carter L.G., Naismith J.H. (2009). The external aldimine form of serine palmitoyltransferase: structural, kinetic, and spectroscopic analysis of the wild-type enzyme and HSAN1 mutant mimics. J. Biol. Chem..

[40] Wang Y., Niu Y., Zhang Z., Gable K., Gupta S.D., Somashekarappa N. (2021). Structural insights into the regulation of human serine palmitoyltransferase complexes. Nat. Struct. Mol. Biol..

[41] Li S., Xie T., Liu P., Wang L., Gong X. (2021). Structural insights into the assembly and substrate selectivity of human SPT-ORMDL3 complex. Nat. Struct. Mol. Biol..

[42] Yard B.A., Carter L.G., Johnson K.A., Overton I.M., Dorward M., Liu H. (2007). The structure of serine palmitoyltransferase; gateway to sphingolipid biosynthesis. J. Mol. Biol..

[43] Raman M.C., Johnson K.A., Clarke D.J., Naismith J.H., Campopiano D.J. (2010). The serine palmitoyltransferase from Sphingomonas wittichii RW1: an interesting link to an unusual acyl carrier protein. Biopolymers.

[44] Wadsworth J.M., Clarke D.J., McMahon S.A., Lowther J.P., Beattie A.E., Langridge-Smith P.R. (2013). The chemical basis of serine palmitoyltransferase inhibition by myriocin. J. Am. Chem. Soc..

[45] Ikushiro H., Takahashi A., Murakami T., Katayama A., Sawai T., Goto H. (2022). Crystal structure of Sphingobacterium multivorum serine palmitoyltransferase complexed with tris(hydroxymethyl)aminomethane. Acta Crystallogr. Sect. F.

[46] Saito S., Murai Y., Usuki S., Yoshida M., Hammam M.A.S., Mitsutake S. (2017). Synthesis of nontoxic fluorous sphingolipids as molecular probes of exogenous metabolic studies for rapid enrichment by fluorous solid phase extraction. Eur. J. Org. Chem..

[47] Yano T., Kagamiyama H. (2001). Directed evolution of ampicillin-resistant activity from a functionally unrelated DNA fragment: a laboratory model of molecular evolution. Proc. Natl. Acad. Sci. U. S. A..

[48] Oue S., Okamoto A., Yano T., Kagamiyama H. (1999). Redesigning the substrate specificity of an enzyme by cumulative effects of the mutations of non-active site residues. J. Biol. Chem..

[49] Kabsch W. (2010). Integration, scaling, space-group assignment and post-refinement. Acta Crystallogr. D Biol. Crystallogr..

[50] Vagin A. (1997). Molrep: an automated program for molecular replacement. J. Appl. Crystallogr..

[51] Winn M.D., Ballard C.C., Cowtan K.D., Dodson E.J., Emsley P., Evans P.R. (2011). Overview of the CCP4 suite and current developments. Acta Crystallogr. D Biol. Crystallogr..

[52] Murshudov (2011). REFMAC5 for the refinement of macromolecular crystal structures. Acta Crystallogr. D Biol. Crystallogr..

[53] Emsley P., Lohkamp B., Scott W.G., Cowtan K. (2010). Features and development of Coot. Acta Crystallogr. Sect. D.

[54] Chen V.B., Arendall W.B., Headd J.J., Keedy D.A., Immormino R.M., Kapral G.J. (2010). MolProbity: All-atom structure validation for macromolecular crystallography. Acta Crystallogr. Sect. D.

[55] Robert X., Gouet P. (2014). Deciphering key features in protein structures with the new ENDscript server. Nucl. Acids Res..

[56] Brünger A.T. (1992). Free R value: a novel statistical quantity for assessing the accuracy of crystal structures. Nature.

