# Supplementary material for: Structural insights into the substrate recognition of serine palmitoyltransferase from Sphingobacterium multivorum
Source: J Biol Chem. 2023 Apr 7;299(5):104684. doi: 10.1016/j.jbc.2023.104684 (PMC10196870; doi:10.1016/j.jbc.2023.104684)
Supplement: Supporting information [file mmc1.docx]

**Supporting Information**

**Structural Insights into the Substrate Recognition of
Serine Palmitoyltransferase from *Sphingobacterium multivorum*.**

Hiroko Ikushiro^1^*, Taiki Murakami^2^, Aya Takahashi^2^, Asuka Katayama^2^,
Taiki Sawai^1^, Haruna Goto^1^, Sajeer Koolath ^3^, Yuta Murai^3^, Kenji Monde^3^,
Ikuko Miyahara^2^, Nobuo Kamiya^2, 4^, and Takato Yano^1^*

^1^ Department of Biochemistry, Faculty of Medicine, Osaka Medical and Pharmaceutical University, 2-7 Daigaku-machi, Takatsuki, Osaka 569-8686, Japan

^2^ Department of Chemistry, Graduate School of Science, Osaka Metropolitan University, 3-3-138 Sugimoto, Sumiyoshi-ku, Osaka, Osaka 558-8585, Japan

^3^ Frontier Research Center for Advanced Material and Life Science, Faculty of Advanced Life Science, Hokkaido University, Kita21 Nishi11, Sapporo, Hokkaido 001-0021, JAPAN

^4^ Research Center for Artificial Photosynthesis, Osaka Metropolitan University, 3-3-138 Sugimoto, Sumiyoshi-ku, Osaka, Osaka 558-8585, Japan

**LIST OF SUPPORTING INFORMATION MATERIAL**

**Figure S1.** Proposed reaction mechanism of *S. multivorum* SPT.

**Figure S2.** Dependence of SPT-catalyzed reaction on substrate concentrations (Michaelis–Menten plot).

**Figure S3.** Amino acid sequence alignment of human SPTLC1 and SPTLC2, and *S. multivorum* SPT.

**Table S1.** Crystallization conditions for crystals from which datasets were collected.

**Supplementary Methods**

**Figure S4.** ^1^H- and ^13^C-NMR analyses of *tert*-butyl (3-(dimethoxyphosphoryl)-2-oxopropyl)carbamate (3a).

**Figure S5. ^1^**H- and ^13^C-NMR analyses of *tert*-butyl (E)-(2-oxoheptadec-3-en-1-yl)carbamate (4a).

**Figure S6.** ^1^H- and ^13^C-NMR analyses of *tert*-butyl (2-oxoheptadecyl)carbamate (5a)**.**

**Figure S7.** ^1^H- and ^13^C-NMR analyses of *1-aminoheptadecan-2-one* (6a).

**Figure S8.** ^1^H- and ^13^C-NMR analyses of *tert*-butyl (S)-(3-oxooctadecan-2-yl)carbamate (5b).

**Figure S9.** ^1^H- and ^13^C-NMR analyses of *(S)*-2-aminooctadecan-3-one (6b).

**Figure S10. ^1^**H- and ^13^C-NMR analyses of *O-(tert-*butyldiphenylsilyl)-*L*-homoserine (7).

**Figure S11.** ^1^H- and ^13^C-NMR analyses of methyl *O-(tert-*butyldiphenylsilyl)-*L*-homoserine (8).

**Figure S12.** ^1^H- and ^13^C-NMR analyses of methyl *N*-(*tert*-butoxycarbonyl)- *O-(tert-*butyldiphenylsilyl)-*L*-homoserine (9).

**Figure S13.** ^1^H- and ^13^C-NMR analyses of *tert*-butyl (*S*)-(5-((*tert*-butyldiphenylsilyl)oxy)-1-(dimethoxyphosphoryl)-2-oxopentan-3-yl)carbamate (10).

**Figure S14.** ^1^H- and ^13^C-NMR analyses of *tert*-butyl (*S,E*)-(1-((*tert*-butyldiphenylsilyl)oxy)-4-oxononadec-5-en-3-yl)carbamate (11).

**Figure S15.** ^1^H- and ^13^C-NMR analyses of *tert*-butyl (*S*)-(1-((*tert*-butyldiphenylsilyl)oxy)-4-oxononadecan-3-yl)carbamate (12).

**Figure S16.** ^1^H- and ^13^C-NMR analyses of (*S*)-3-amino-1-hydroxynonadecan-4-one （and (3*S*)-3-amino-2-pentadecyltetrahydrofuran-2-ol） (13).

**Figure S1. Proposed reaction mechanism of *S. multivorum* SPT.**

At the active site of SPT, PLP forms an aldimine with the ε-amino group of Lys244 (internal aldimine, **I**). The internal aldimine undergoes transaldimination with the first substrate L-Ser to yield the PLP–L-Ser aldimine (external aldimine, **IIa**). As shown in the crystal structure of the external aldimine in the SPT–L-Ser binary complex (Fig. 5), the conformation of the external aldimine, which is brought about by the hydrogen bond between L-Ser and the N_ε2_ of His138, is unfavorable for the α-deprotonation of L-Ser. Binding of the second substrate palmitoyl-CoA induces changes in the orientation of the L-Ser moiety of the external aldimine to cancel the interaction with the side chain of His138, and the carbonyl group of palmitoyl-CoA binds to Nε2 of His138 (external aldimine, **IIb**). This causes the Cα–H bond to be nearly perpendicular to the imine–pyridine plane (**IIb**), which is favorable for the α-deprotonation to form the quinonoid intermediate (**IIIa**). The carbanionic Cα of **IIIa** attacks palmitoyl-CoA (Claisen-type condensation), and CoASH is released from **IIIb** to generate a condensation product **(IV**), which, by decarboxylation, yields the second quinonoid intermediate (**V**). Protonation at Cα of **V** gives the external aldimine of PLP-KDS (**VI**). Finally, release of KDS regenerates the internal aldimine (**I**).

**Figure S2. Kinetic analysis of SPT.**

The reactions were carried out in the presence of 1 mM PalCoA as described in Experimental procedures. Apparent rate constants, obtained by dividing the rate of the LCB production by the concentration of the enzyme, were plotted against concentrations of amino acid substrate, L-Ser (A), L-Hse (B), L-Ala (C), or glycine (D). The plots were fitted to the equation of the Michaelis–Menten mechanism, v = *V*_max_[S]/(*K*_m_+[S]), by nonlinear regression using the software Igor Pro (ver 6.37, WaveMetrics, Lake Oswego, OR), and the $K_{m}^{app}$and $k_{cat}^{app}$ of SPT for each amino acid substrate were calculated. Solid line represents a theoretical curve based on the above equation. Each data point is a mean with the error bar showing standard deviation from at least three independent experiments.

**Figure S3. Amino acid sequence alignments between human SPTLC1 or SPTLC2 and *S. multivorum* SPT**.

The alignment was constructed by the structure-based amino acid sequence alignment between human SPTLC1 and the *S. multivorum* SPT(A) or SPTLC2 and the *S. multivorum* SPT (B). The conserved amino acid residues are shown on a black background, and the residues with high similarity are indicated by boxes. The disease-related amino acid residues were categorized into three groups: near the PLP-binding site (*green*), distributed sporadically on SPTLC1 (*red*), and located on the SPT/ORM protein interface (*blue*). The secondary structures of the *S. multivorum* SPT are shown at the top, and those of human SPT subunit proteins are shown at the bottom. The figure was prepared with ESPript (1) by applying the %Equivalent scoring matrix.

**Table S1. Crystallization conditions for crystals from which datasets were collected**

| Crystal | Primary precipitant | Salt | Buffer  (pH) | Note |
| --- | --- | --- | --- | --- |
| Ligand-free | 15.0%(w/v) PEG4000 | 200 mM sodium acetate | 100 mM Tris (pH 8.5) | Soaked in 100 mM Tricine (pH 8.5), 200 mM sodium acetate, 15.0%(w/v) PEG4000 for 90 min |
| L-Ser-complex | 15.5%(w/v) PEG4000 | 200 mM sodium acetate | 100 mM Tris (pH 8.5) | Soaked in 100 mM Tricine (pH 8.5), 200 mM sodium acetate, 15.5%(w/v) PEG4000, 285 mM L-Ser for 5 min |
| L-Hse-complex | 16.5%(w/v) PEG4000 | 200 mM sodium acetate | 100 mM Tris (pH 8.5) | Soaked in 100 mM Tricine (pH 8.5), 200 mM sodium acetate, 16.5%(w/v) PEG4000, 235 mM L-Hse for 5 min |
| Gly-complex | 15.0%(w/v) PEG4000 | 200 mM sodium acetate | 100 mM Tris (pH 8.5) | Soaked in 100mM Tricine (pH 8.5), 200 mM sodium acetate, 15.0%(w/v) PEG4000, 266 mM Gly for 40 min |
| L-Ala-complex | 15.0%(w/v) PEG4000 | 200 mM sodium acetate | 100 mM Tris (pH 8.5) | Soaked in 100mM Tricine (pH 8.5), 200 mM sodium acetate, 15.0%(w/v) PEG4000, 213 mM L-Ala for 5 min |
| L-Thr-complex | 16.5%(w/v) PEG4000 | 200 mM sodium acetate | 100 mM Tris (pH 8.5) | Soaked in 100 mM Tricine (pH 8.5), 200 mM sodium acetate, 16.5%(w/v) PEG4000, 172 mM L-Thr for 5 min |

**Supplementary Methods**

***Synthesis of authentic standards, 1-aminoheptadecan-2-one, (S)-2-aminooctadecan-3-one, and (S)-3-amino-1-hydroxynonadecan-4-one.***

3-keto LCB derived from Gly or L-Ala (**6a, b**) could be constructed with commercial items; *N*- *tert*-butoxycarbonyl (Boc) protected Gly or L-Ala and 1-tetradecanal as summarized in Scheme 1. Initially, *N*-Boc protected Gly or L-Ala (**1**) was subjected to protection of the carboxy group with methyl ester (**2**). Compounds **2** were transformed into the corresponding β-ketophosphonates (**3**) by treatment with excess lithium dimethyl methylphosphonate at –78 °C in moderate yield (52–76%). Next, compounds **3** and 1-tetradecanal underwent trans-selective double-bond formation through a modified HWE olefination with LiCl and triethylamine in THF to give 3-keto-sphingosines (**4**) in good yield (64–92%). Subsequently, compounds **4** were subjected to reduction of the olefin to give compounds **5**, then deprotection of Boc group with TFA/DCM to produce desired 3-keto sphinganines (**6**) in high yield (88–91%).

Scheme 1. Synthesis of glycine and alanine type 3-keto sphinganines

On the other hand, we first tried to prepare homoserine type 3-keto sphinganine (**13**) as previously reported (46). However, 1,3-oxazinane intermediate compound could not be synthesized. Therefore, the synthetic method was changed to overcome the requirement to protect homoserine. Outline of the synthesis was summarized in Scheme 2. First, the primary hydroxy group of homoserine was protected with a *tert*-butyldiphenylsilyl (TBDPS) (**7**) and then the carboxyl group was with trimethylsilyl (TMS)-diazomethane to give compound **8** in quantitative yield (96%). Subsequently, the amino group was protected with Boc without purification to obtain compound **9** in good yield (75%). After constructing β-ketophosphonate (**10**), HWE olefination (**11**) and reduction (**12**) were conducted in moderate yield (53–88%). Finally, both protection groups were deprotected by TFA to give the desired 3-keto sphinganine (**13**). Compound **13** should form a cyclized compound at equilibrium; a 5-membered ring is thermodynamically more stable than a linear form.

Scheme 2. Synthesis of homoserine type 3-keto sphinganine

***Synthesis of tert-butyl (3-(dimethoxyphosphoryl)-2-oxopropyl) carbamate (3a)***

To a stirred solution of dimethyl methylphosphonate (860 µL, 7.93 mmol) and THF, *n*-BuLi in hexane (3.2 mL, 7.93 mmol) was added dropwise at –78 ºC . After 30 minutes, compound **3** (500 mg, 2.64 mmol) in 10 mL THF was added to the reaction mixture dropwise and stirred overnight at –78 ºC. Then the reaction mixture was quenched with 10% citric acid. Next, the solvent was removed under reduced pressure and the resulting residue was taken up in ethyl acetate (EtOAc). The organic layer was separated, and the aqueous layer was further extracted with EtOAc. The combined organic layers were dried with MgSO_4_ before being concentrated under reduced pressure. Then the residue was purified by silica gel column chromatography (n-hexane/EtOAc = 1:1) to yield compound **4** (383 mg, 52%).

^1^H NMR (500 MHz, CDCl_3_) δ 5.40 (br. s., 1H), 4.04 (d, J = 5.13 Hz, 2H), 3.70 (s, 3H), 3.72 (s, 3H), 3.09 (s, 1H), 3.04 (s, 1H), 1.36 (s, 9H). ^13^C NMR (126 MHz, CDCl_3_) δ 198.0, 155.6, 79.8, 53.1, 51.2, 39.0, 38.0, 28.2. HRMS (m/z): [M+H]^+^ calculated for C_10_H_20_NO_6_P: 282.1101 found 282.1098.

***Synthesis of tert-butyl (E)-(2-oxoheptadec-3-en-1-yl) carbamate (4a)***

To a stirred solution of β-ketophosphonate (363 mg, 1.29 mmol) and K_2_CO_3_ (713 mg, 5.16 mmol) in THF/H_2_O (1:1), tetradecanal (274 mg, 1.29 mmol) was added at 0 ºC. The reaction mixture was stirred overnight at room temperature. The reaction mixture was acidified with citric acid solution and extracted with EtOAc. The organic phase was washed with NaHCO_3_ and brine and dried with MgSO_4_, and the solvents were evaporated. The crude product was purified by silica gel column chromatography (*n*-hexane/EtOAc = 10:1) to yield compound **5** (303 mg, 64%).

^1^H NMR (500 MHz, CDCl_3_) δ 6.95 (td, J = 6.99, 15.82 Hz, 1H), 6.11 (d, J = 15.88 Hz, 1H), 5.40 (br. s., 1H), 4.13 - 4.26 (m, 2H), 2.23 (q, J = 6.68 Hz, 2H), 1.45 (s, 9H), 1.23 - 1.30 (m, 22H), 0.88 (t, J = 6.84 Hz, 3H). ^13^C NMR (126 MHz, CDCl_3_) δ 194.4, 155.7, 149.5, 127.3, 83.6, 48.3, 34.0, 32.7, 31.9, 29.6, 29.3, 28.3, 27.9, 24.7, 22.7, 14.1. ESI-MS(m/z): 390.33 [M+Na]^+^.

***Synthesis of tert-butyl (2-oxoheptadecyl) carbamate (5a)***

To a solution of compound **5** (75 mg, 0.20 mmol) in EtOAc (4 ml) was added 10 % palladium-activated carbon (Pd/C) (43 mg, 0.04 mmol). The reaction mixture was stirred overnight at room temperature under H_2_ atmosphere. Pd/C was filtered off, and the filtrate was concentrated under reduced pressure. The residue was purified by silica gel column chromatography using (n-hexane/ EtOAc = 10:1) to give compound **6** (55 mg, 74%).

^1^H NMR (500 MHz, CDCl_3_) δ 5.26 (br. s., 1H), 4.01 (d, J = 3.91 Hz, 2H), 2.41 (t, J = 7.45 Hz, 2H), 1.58 - 1.65 (m, 2H), 1.44 (s, 9H), 1.23 – 1.32 (m, 24H), 0.86 (t, J = 6.84 Hz, 3H). ^13^C NMR (126 MHz, CDCl_3_) δ 205.9, 155.6, 79.8, 50.2, 40.1, 33.9, 31.9, 29.7, 29.7, 29.7, 29.6, 29.4, 29.3, 29.2, 29.1, 28.3, 24.7, 23.7, 22.7, 14.1. HRMS (m/z): [M+H]^+^ calculated for C_22_H_43_NO_3_: 370.3315 found 370.3309.

***Synthesis of 1-aminoheptadecan-2-one (6a)***

To a stirred solution of compound **6** (44 mg, 0.12 mmol) in CH_2_Cl_2_ (1 ml) cooled at 0 °C, TFA (1 ml) was added dropwise. The resulting mixture was stirred for 1.5 h at room temperature. The reaction was monitored by TLC. After the reaction mixture was concentrated under vacuum, the resulting crude product was purified by silica gel column chromatography (CHCl_3_/MeOH = 96:4) to yield compound **7** (28 mg, 88%).

^1^H NMR (500 MHz, CD_3_OD) δ 3.94 (s, 2H), 2.54 (t, J = 7.45 Hz, 2H), 1.62 (t, J = 6.96 Hz, 2H), 1.27 - 1.35 (m, 24H), 0.90 (t, J = 6.84 Hz, 3H). ^13^C NMR (126 MHz, CD_3_OD) δ 204.4, 48.1, 40.7, 33.2, 31.0, 30.9, 30.8, 30.7, 30.6, 30.4, 30.3, 24.4, 23.9, 14.6. HRMS (m/z): [M+H]^+^ calculated for C_17_H_35_NO: 270.2791 found 270.2786.

***Synthesis of methyl (tert-butoxycarbonyl)-L-alaninate (2b)***

To a stirred solution of L-Boc-Alanine (500 mg, 2.64 mmol) and Na_2_CO_3_ (560 mg, 2.64 mmol) in DMF, CH_3_I (700 µL, 10.6 mmol) was added dropwise. The reaction mixture was stirred for 20 h at room temperature. The mixture was filtered, and the resulting filtrate were extracted by EtOAc and washed with brine. The combined organic layers were dried with MgSO_4_ before being concentrated under reduced pressure. The crude product was purified by silica gel chromatography (*n*-hexane/EtOAc = 9:1) to yield compound **2** (530 mg, 98%).

***Synthesis of tert-butyl (S)-(4-(dimethoxyphosphoryl)-3-oxobutan-2-yl) carbamate (3b)***

To a stirred solution of dimethyl methyl phosphonate (795 µL, 7.43 mmol) and THF, *n*-BuLi in n-hexane (3.0 mL, 7.43 mmol) was added dropwise at –78 ºC. After stirred for 30 minutes, compound **2** (503 mg, 2.48 mmol) in 10 mL THF was added to the reaction mixture dropwise and stirred overnight at –78 ºC. Then the reaction mixture was quenched with 10% citric acid. Next, the solvent was removed under reduced pressure and the resulting residue was taken up in EtOAc. The organic layer was separated, and the aqueous layer was further extracted with EtOAc. The combined organic layers were dried with MgSO_4_ before being concentrated under reduced pressure. Then the residue was purified by silica gel column chromatography (*n*-hexane/EtOAc = 1:1) to yield compound **3** (453 mg, 62%).

***Synthesis of tert-butyl (S, E) -(3-oxooctadec-4-en-2-yl) carbamate (4b)***

To a stirred solution of tetradecanal (577 mg, 2.72 mmol), β-ketophosphonate (400 mg, 1.36 mmol), and LiCl (172 mg, 4.07 mmol) in THF, triethyl amine (566 µL, 4.07 mmol) was added dropwise at 0 ºC. The reaction mixture was stirred overnight at room temperature. The reaction mixture was acidified with citric acid solution and extracted with diethyl ether (Et_2_O). The organic phase was washed with NaHCO_3_ and brine, and dried with MgSO_4_, and the solvents were evaporated. The crude product was purified by silica gel column chromatography (*n*-hexane/ EtOAc = 10:1) to yield compound **4** (402 mg, 77%).

***Synthesis of tert-butyl (S)-(3-oxooctadecan-2-yl) carbamate (5b)***

To a stirred solution of compound **4** (109 mg, 0.29 mmol) in EtOAc (5 ml) was added 10% Pd/C (62 mg, 0.058 mmol). The reaction mixture was stirred overnight at room temperature under H_2_ atmosphere. Pd/C was filtered off and the filtrate was concentrated under reduced pressure. The residue was purified by silica gel column chromatography using (*n*-hexane/ EtOAc = 10:1) to yield compound **5** (84 mg, 76%).

^1^H NMR (500 MHz, CDCl_3_) δ 5.24 (d, J = 7.46 Hz, 1H), 4.30 (m, 1H), 2.38 - 2.55 (m, 2H), 1.58 (m, 2H), 1.43 (s, 9H), 1.29 - 1.33 (m, 3H), 1.24 – 1.28 (m, 24H), 0.87 (t, J = 6.96 Hz, 3H). ^13^C NMR (126 MHz, CDCl_3_) δ 209.7, 155.2, 79.6, 57.0, 39.2, 31.9, 29.7, 29.6, 29.4, 29.3, 29.2, 28.3, 23.6, 22.7, 17.9, 14.1. HRMS (m/z): [M+H]^+^ calculated for C_23_H_45_NO_3_: 384.3472, found 384.3460.

***Synthesis of (S)-2-aminooctadecan-3-one (6b)***

A solution of compound 5 (84 mg, 0.22 mmol) in CH_2_Cl_2_ (2 ml) was cooled to 0 °C. Then TFA (2 ml) was added dropwise to the reaction mixture. The resulting mixture was stirred for 1.5 h at room temperature. The reaction was monitored by TLC. The reaction mixture was concentrated, and the resulting crude product was purified by silica gel column chromatography (CHCl_3_/MeOH/TEA = 95:4:1) to yield compound **6** (35 mg, 56%).

^1^H NMR (500 MHz, CDCl_3_) δ 8.19 (br. s., 2H), 4.14 - 4.22 (m, 1H), 2.41 - 2.60 (m, 2H), 1.53 – 1.64 (m, 5H), 1.26 (br. s., 24H), 0.89 (t, J = 6.84 Hz, 3H). ^13^C NMR (126 MHz, CDCl_3_) δ 206.5, 54.9, 38.4, 31.9, 29.7, 29.6, 29.5, 29.4, 29.3, 29.0, 23.1, 22.7, 15.4, 14.1. HRMS (m/z): [M+H]^+^ calculated for C18H37NO: 284.2947, found 284.2947.

***Synthesis of O-(tert-butyldiphenylsilyl)-L-homoserine (7)***

To a stirred solution of L-homoserine (1.0 g, 8.39 mmol) in acetonitrile (CH_3_CN) (20 mL) at 0 ºC were added DBU (1.5 mL, 10.07 mmol) and *tert*-butyldiphenylchlorosilane (TBDPSCl) (2.6 mL, 10.07 mmol). The reaction mixture was stirred for 20 h at room temperature. Then the reaction mixture was filtered and concentrated under reduced pressure. The obtained residue was washed with CH_3_CN and then Et_2_O. Compound **7** (1.4 g, crude) was obtained.

^1^H NMR (500 MHz, CD_3_OD) δ 7.67 - 7.72 (m, 4H), 7.39 - 7.47 (m, 6H), 3.87 - 3.93 (m, 2H), 3.75 (dd, J = 4.89, 7.57 Hz, 1H), 2.20 - 2.31 (m, 1H), 1.90 – 2.08 (m, 1H), 1.06 (s, 9H). ^13^C NMR (126 MHz, CD_3_OD) δ 183.2, 135.3, 129.7, 129.6, 127.5, 61.2, 53.0, 48.1, 48.0, 47.8, 47.6, 47.4, 47.3, 47.1, 33.2, 26.0, 18.5. HRMS (m/z): [M+H]^+^ calculated for C_21_H_27_NO_3_Si: 358.1753 found 358.1784.

***Synthesis of methyl O-(tert-butyldiphenylsilyl)-L-homoserinate (8)***

To a stirred solution of compound **7** (645 mg, 1.80 mmol) in methanol/toluene (7.5 mL:25 mL) was added TMS-diazomethane in hexane (15 mL, 9.02 mmol) at room temperature. Then the reaction mixture was stirred overnight. The solution was concentrated under reduced pressure. Then the residue was purified by silica gel column chromatography (CHCl_3_/MeOH/TEA = 50:5:3) to yield compound **8** (638 g, 96%).

^1^H NMR (500 MHz, CDCl_3_) δ 7.70 (d, J = 7.33 Hz, 4H), 7.34 - 7.43 (m, 6H), 3.77 - 3.88 (m, 2H), 3.72 (dd, J = 4.76, 7.94 Hz, 1H), 3.67 (s, 3H), 2.01 – 2.10 (m, 1H), 1.73 - 1.81 (m, 1H), 1.08 (s, 9H). ^13^C NMR (126 MHz, CDCl3) δ 176.4, 135.6, 133.6, 129.7, 127.7, 60.7, 51.9, 51.8, 37.2, 26.9, 19.2. HRMS (m/z): [M+H]^+^ calculated for C_21_H_29_NO_3_Si: 372.1989 found 372.1946.

***Synthesis of methyl N-(tert-butoxycarbonyl)-O-(tert-butyldiphenylsilyl)-L-homoserinate (9)***

To a solution of compound **8** (635 mg, 1.71 mmol) in acetone/H_2_O (5mL:5mL) were added TEA (473 µL, 3.4 mmol) and Boc_2_O (445 mg, 2.04 mmol). Then the reaction mixture was stirred for 1.5 h at room temperature. The reaction mixture was concentrated under reduced pressure and extracted with EtOAc. The combined organic layer was washed with brine and dried by MgSO_4_. Then the residue was purified by silica gel column chromatography (*n*-hexane/EtOAc = 6:1) to yield compound **9** (600 mg, 75%).

^1^H NMR (500 MHz, CDCl_3_) δ 7.68 - 7.71 (m, 4H), 7.39 - 7.47 (m, 6H), 5.83 (d, J = 7.57 Hz, 1H), 4.44 - 4.52 (m, 1H), 3.74 - 3.78 (m, 2H), 3.72 (s, 3H), 2.09 - 2.17 (m, 1H), 1.94 - 2.03 (m, 1H), 1.47 (s, 9H), 1.10 (s, 9H). ^13^C NMR (126 MHz, CDCl_3_) δ 173.0, 155.6, 135.6, 135.6, 129.8, 127.8, 79.5, 61.1, 52.4, 52.2, 33.7, 28.4, 26.9, 26.8, 22.7, 19.1. HRMS (m/z): [M+H]^+^ calculated for C_26_H_37_NO_5_Si: 472.2513 found 472.2481.

***Synthesis of tert-butyl (S)-(5-((tert-butyldiphenylsilyl)oxy)-1-(dimethoxyphosphoryl)-2-oxopentan-3-yl)carbamate (10)***

To a stirred solution of dimethyl methylphosphonate (580 µL, 5.36 mmol) and THF, *n*-BuLi in hexane (2.1 mL, 5.36 mmol) was added dropwise at –78 ºC. After stirred for 30 minutes, compound **9** (842 mg, 1.79 mmol) in 15 mL THF was added to the reaction mixture dropwise and stirred overnight at –78 ºC. Then the reaction mixture was quenched with 10% citric acid. Next, the solvent was removed under reduced pressure and the resulting residue was taken up in EtOAc. The organic layer was separated, and the aqueous layer was further extracted with EtOAc. The combined organic layers were dried with MgSO_4_ before being concentrated under reduced pressure. Then the residue was purified by silica gel column chromatography (*n*-hexane/EtOAc = 1:1) to yield compound **10** (889 mg, 88%).

^1^H NMR (500 MHz, CDCl_3_) δ 7.62 - 7.67 (m, 4H), 7.35 - 7.46 (m, 6H), 5.86 (d, J = 6.35 Hz, 1H), 4.40 – 4.47 (m, 1H), 3.77 - 3.79 (m, 3H), 3.75 - 3.77 (m, 3H), 3.68 - 3.75 (m, 2H), 3.31 - 3.41 (m, 1H), 3.11 - 3.21 (m, 1H), 2.09 - 2.19 (m, 1H), 1.87 - 1.97 (m, 1H), 1.43 (s, 9H), 1.05 (s, 9H). ^13^C NMR (126 MHz, CDCl_3_) δ 213.2, 183.7, 135.5, 133.0, 129.8, 127.8, 79.9, 60.9, 59.2, 53.1, 38.1, 32.5, 28.3, 26.8, 19.1. HRMS (m/z): [M+H]+ calculated for C_28_H_42_NO_7_PSi: 564.2541 found 564.2509.

***Synthesis of tert-butyl (S,E)-(1-((tert-butyldiphenylsilyl)oxy)-4-oxononadec-5-en-3-yl)carbamate (11)***

To a stirred solution of β-ketophosphonate (857 mg, 1.52 mmol) and K_2_CO_3_ (840 mg, 6.08 mmol) in THF/H_2_O (1:1), tetradecanal (387 mg, 1.82 mmol) was added at 0 ºC. The reaction mixture was stirred overnight at room temperature. The reaction mixture was acidified with citric acid solution and extracted with EtOAc. The organic phase was washed with NaHCO_3_ and brine and dried with MgSO_4_, and the solvents were evaporated. The crude product was purified by silica gel column chromatography (*n*-hexane/ EtOAc = 10:1) to yield compound **11** (520 mg, 53%).

^1^H NMR (500 MHz, CDCl_3_) δ 7.62 - 7.70 (m, 4H), 7.33 - 7.48 (m, 6H), 7.01 (td, J = 6.90, 15.76 Hz, 1H), 6.23 (d, J = 15.64 Hz, 1H), 5.66 (d, J = 7.57 Hz, 1H), 4.66 - 4.74 (m, 1H), 3.64 - 3.77 (m, 2H), 2.21 (q, J = 6.92 Hz, 2H), 2.01 - 2.13 (m, 1H), 1.75 - 1.84 (m, 1H), 1.43 (s, 9H), 1.26 - 1.33 (m, 22H), 1.06 (s, 9H), 0.88 (t, J = 7.01 Hz, 3H). ^13^C NMR (126 MHz, CDCl_3_) δ 198.2, 162.8, 161.1, 135.5, 134.8, 133.3, 129.7, 127.7, 79.4, 60.6, 55.8, 34.5, 32.7, 31.9, 29.7, 29.6, 29.5, 29.4, 29.3, 29.2, 29.1, 28.3, 27.9, 26.8, 24.7, 22.7, 19.1, 14.1. HRMS (m/z): [M+H]^+^ calculated for C_40_H_63_NO_4_Si: 650.4599 found 650.4568.

***Synthesis of tert-butyl (S)-(1-((tert-butyldiphenylsilyl)oxy)-4-oxononadecan-3-yl)carbamate (12)***

To a solution of compound **11** (143 mg, 0.22 mmol) in EtOAc (4 ml) was added 10% Pd/C (47 mg, 0.044 mmol). The reaction mixture was stirred overnight at room temperature under H_2_ atmosphere. Pd/C was filtered off and the filtrate was concentrated under reduced pressure. The residue was purified by silica gel column chromatography using (*n*-hexane/ EtOAc = 10:1) to yield compound **12** (119 mg, 83%).

^1^H NMR (500 MHz, CDCl_3_) δ 7.63 - 7.68 (m, 4H), 7.37 - 7.47 (m, 6H), 5.73 (d, J = 6.84 Hz, 1H), 4.37 (d, J = 4.64 Hz, 1H), 3.65 - 3.75 (m, 2H), 2.52 (t, J = 7.45 Hz, 2H), 2.01 - 2.10 (m, 1H), 1.82 – 1.91 (m, 1H), 1.51 - 1.61 (m, 2H), 1.44 (s, 9H), 1.26 (br. s., 24H), 1.06 (s, 9H), 0.89 (t, J = 6.96 Hz, 3H). ^13^C NMR (126 MHz, CDCl_3_) δ 209.8, 162.8, 161.1, 135.5, 129.8, 127.7, 79.5, 60.8, 58.1, 39.5, 31.9, 29.7, 29.7, 29.6, 29.6, 29.6, 29.6, 29.5, 29.4, 29.4, 29.3, 29.3, 29.2, 29.2, 29.1, 28.3, 26.8, 24.7, 23.5, 22.7, 19.0, 14.1. HRMS (m/z): [M+H]^+^ calculated for C_40_H_65_NO_4_Si: 652.4755 found 652.4721.

***Synthesis of (S)-3-amino-1-hydroxynonadecan-4-one (13)***

To compound **12** (42 mg, 0.064 mmol) was added TFA (1 ml) at 0 °C. The resulting mixture was stirred for 2 h at room temperature. Then, MeOH (500 µL) was added to the reaction mixture and stirred for 48 h at room temperature. The reaction mixture was concentrated under vacuum. The resulting crude product was purified by silica gel column chromatography (CHCl_3_/MeOH = 10:1) to yield compound **13** (18 mg, 90%). At equilibrium, the compound **13** undergo formation of the cyclized compound.

^1^H NMR (500 MHz, CD_3_OD) δ 4.23 (dd, J = 3.66, 8.06 Hz, 1H), 4.04 – 4.11 (m, 1H), 3.85 – 3.92 (m, 1H), 3.70 - 3.82 (m,2H), 3.52 – 3.57 (m, 1H), 2.67 – 2.73 (m, 1H), 2.54 – 2.63 (m, 1H), 2.31 - 2.44 (m, 1H), 2.18 - 2.26 (m, 1H), 1.95 - 2.10 (m, 1H), 1.72 - 1.85 (m, 1H), 1.59 - 1.67 (m, 2H), 1.41 – 1.49 (m, 1H), 1.30 - 1.35 (m, 24H), 0.88 - 0.95 (t, J = 6.84 Hz ,3H). ^13^C NMR (126 MHz, CD_3_OD) δ 209.3, 121.1, 60.9, 58.1, 46.5, 37.7, 33.3, 31.7, 30.3, 29.4, 29.3, 29.2, 29.1, 28.8, 24.6, 22.3, 13.0. HRMS (m/z): [M+H]^+^ calculated for C_19_H_39_NO_2_: 314.3053 found 314.3009.

**Refferences**

1. Robert, X., and Gouet, P. (2014) Deciphering key features in protein structures with the new ENDscript server. *Nucleic Acids Res.* **42**, W320-324

2. Saito, S., Murai, Y., Usuki, S., Yoshida, M., Hammam, M. A. S., Mitsutake, S., Yuyama, K., Igarashi, Y., and Monde, K. (2017) Synthesis of Nontoxic Fluorous Sphingolipids as Molecular Probes of Exogenous Metabolic Studies for Rapid Enrichment by Fluorous Solid Phase Extraction. *European J. Org. Chem.* **2017**, 1045-1051

^1^H-NMR

^13^C-NMR

**Figure S4. ^1^H- and ^13^C-NMR analyses of *tert*-butyl (3-(dimethoxyphosphoryl)-2-oxopropyl)carbamate (3a).**

^1^H-NMR

^13^C-NMR

**Figure S5. ^1^H- and ^13^C-NMR analyses of *tert*-butyl (E)-(2-oxoheptadec-3-en-1-yl)carbamate (4a).**

^1^H-NMR

^13^C-NMR

**Figure S6. ^1^H- and ^13^C-NMR analyses of *tert*-butyl (2-oxoheptadecyl)carbamate (5a).**

^1^H-NMR

^13^C-NMR

**Figure S7. ^1^H- and ^13^C-NMR analyses of *1-aminoheptadecan-2-one* (6a).**

^1^H-NMR

^13^C-NMR

**Figure S8. ^1^H- and ^13^C-NMR analyses of *tert*-butyl (S)-(3-oxooctadecan-2-yl)carbamate (5b).**

^1^H-NMR

^13^C-NMR

**Figure S9. ^1^H- and ^13^C-NMR analyses of *(S)*-2-aminooctadecan-3-one (6b).**

^1^H-NMR

^13^C-NMR

**Figure S10. ^1^H- and ^13^C-NMR analyses of *O-(tert-*butyldiphenylsilyl)-*L*-homoserine (7).**

^1^H-NMR

^13^C-NMR

**Figure S11. ^1^H- and ^13^C-NMR analyses of methyl *O-(tert-*butyldiphenylsilyl)-*L*-homoserine (8).**

^1^H-NMR

^13^C-NMR

**Figure S12. ^1^H- and ^13^C-NMR analyses of methyl *N*-(*tert*-butoxycarbonyl)- *O-(tert-*butyldiphenylsilyl)-*L*-homoserine (9).**

^1^H-NMR

^13^C-NMR

**Figure S13. ^1^H- and ^13^C-NMR analyses of *tert*-butyl (*S*)-(5-((*tert*-butyldiphenylsilyl)oxy)-1-(dimethoxyphosphoryl)-2-oxopentan-3-yl)carbamate (10).**

^1^H-NMR

^13^C-NMR

**Figure S14. ^1^H- and ^13^C-NMR analyses of *tert*-butyl (*S,E*)-(1-((*tert*-butyldiphenylsilyl)oxy)-4-oxononadec-5-en-3-yl)carbamate (11).**

^1^H-NMR

^13^C-NMR

**Figure S15. ^1^H- and ^13^C-NMR analyses of *tert*-butyl (*S*)-(1-((*tert*-butyldiphenylsilyl)oxy)-4-oxononadecan-3-yl)carbamate (12).**

^1^H-NMR

^13^C-NMR

**Figure S16. ^1^H- and ^13^C-NMR analyses of (*S*)-3-amino-1-hydroxynonadecan-4-one （and (3*S*)-3-amino-2-pentadecyltetrahydrofuran-2-ol） (13).**

**Table S1. Crystallization conditions for crystals from which datasets were collected**

| Crystal | Primary precipitant | Salt | Buffer  (pH) | Note |
| --- | --- | --- | --- | --- |
| Ligand-free | 15% (v/v) PEG4000 | 200mM sodium acetate | 100 mM Tris (pH 8.5) | Soaked in 100 mM Tricine (pH 8.5), 200 mM sodium acetate, 15%(v/v) PEG4000 for 90 min |
| L-Ser-complex | 15.5 % (v/v) PEG4000 | 200mM sodium acetate | 100 mM Tris (pH 8.5) | Soaked in 100 mM Tricine (pH 8.5), 200 mM sodium acetate, 15.5%(v/v) PEG4000, 285 mM L-Ser for 5 min |
| L-Hse-complex | 16.5% (v/v) PEG4000 | 200mM sodium acetate | 100 mM Tris (pH 8.5) | Soaked in 100 mM Tricine (pH 8.5), 200 mM sodium acetate, 16.5%(v/v) PEG4000, 235 mM L-Hse for 5 min |
| Gly-complex | 15% (v/v) PEG4000 | 200mM sodium acetate | 100 mM Tris (pH 8.5) | Soaked in 100mM Tricine (pH 8.5), 200 mM sodium acetate, 15% (v/v) PEG4000, 266 mM Gly for 40 min |
| L-Ala-complex | 15% (v/v) PEG4000 | 200mM sodium acetate | 100 mM Tris (pH 8.5) | Soaked in 100mM Tricine (pH 8.5), 200 mM sodium acetate, 15.0% (v/v) PEG4000, 213 mM L-Ala for 5 min |
| L-Thr-complex | 16.5% (v/v) PEG4000 | 200mM sodium acetate | 100 mM Tris (pH 8.5) | Soaked in 100 mM Tricine (pH 8.5), 200 mM sodium acetate, 16.5% (v/v) PEG4000, 172 mM L-Thr for 5 min |
